# Nerve injury increases native Ca_V_2.2 trafficking in dorsal root ganglion mechanoreceptors

**DOI:** 10.1097/j.pain.0000000000002846

**Published:** 2022-12-15

**Authors:** Manuela Nieto-Rostro, Ryan Patel, Anthony H. Dickenson, Annette C. Dolphin

**Affiliations:** Department of Neuroscience, Physiology and Pharmacology, University College London, London, United Kingdom

**Keywords:** Neuropathic pain, Nerve injury, DRG neuron, N-type calcium channel, α_2_δ-1 subunit, Trafficking, Immunocytochemistry

## Abstract

Neuronal N-type (Ca_V_2.2) voltage-gated calcium channels are essential for neurotransmission from primary afferent terminals in the dorsal horn. In this study, we have used a knockin mouse containing Ca_V_2.2 with an inserted extracellular hemagglutinin tag (Ca_V_2.2_HA), to visualise the pattern of expression of endogenous Ca_V_2.2 in dorsal root ganglion (DRG) neurons and their primary afferents in the dorsal horn. We examined the effect of partial sciatic nerve ligation (PSNL) and found an increase in Ca_V_2.2_HA only in large and medium dorsal root ganglion neurons and also in deep dorsal horn synaptic terminals. Furthermore, there is a parallel increase in coexpression with GFRα1, present in a population of low threshold mechanoreceptors, both in large DRG neurons and in their terminals. The increased expression of Ca_V_2.2_HA in these DRG neurons and their terminals is dependent on the presence of the auxiliary subunit α_2_δ-1, which is required for channel trafficking to the cell surface and to synaptic terminals, and it likely contributes to enhanced synaptic transmission at these synapses following PSNL. By contrast, the increase in GFRα1 is not altered in α_2_δ-1–knockout mice. We also found that following PSNL, there is patchy loss of glomerular synapses immunoreactive for Ca_V_2.2_HA and CGRP or IB4, restricted to the superficial layers of the dorsal horn. This reduction is not dependent on α_2_δ-1 and likely reflects partial deafferentation of C-nociceptor presynaptic terminals. Therefore, in this pain model, we can distinguish 2 different events affecting specific DRG terminals, with opposite consequences for Ca_V_2.2_HA expression and function in the dorsal horn.

## Abstract 2

Supplemental Digital Content is Available in the Text.

Using a Ca_V_2.2_HA knockin mouse, it was found that following nerve injury, Ca_V_2.2_HA increases in medium/large dorsal root ganglion neurons and deep dorsal horn, associated with GFRα1.

## 1. Introduction

N-type calcium channels play an essential role in primary afferent neurotransmission in the spinal cord dorsal horn.^11,36^ Indeed, these channels were first identified in dorsal root ganglion (DRG) neurons.^20,42^ The use of selective peptide blockers including ω-conotoxin GVIA^6,46^ has furthered understanding of the importance of N-type channels^23,24^ and their distribution.^1,22^ Their targeting for chronic pain therapy is also well-established.^51,57^ Although clinical use of ziconotide (ω-conotoxin MVIIA) is limited because of its intrathecal route of administration and side effects, nevertheless, it validates the role of N-type channels in pain pathophysiology and pharmacotherapy.^45^

Ca_V_2.2 channels form a complex with auxiliary β and α_2_δ subunits, which are important for channel trafficking and function.^18^ The α_2_δ-1 isoform is prominent in primary afferent pathways and is up-regulated following neuropathic injury.^4,34,40^ Indeed, genetic ablation of either Ca_V_2.2^28,47^ or α_2_δ-1^44^ suppresses various sensory modalities in chronic pain models.

Despite the importance of Ca_V_2.2 channels in primary afferent transmission in nociceptive pathways, examination of their distribution and trafficking as well as altered expression following nerve injury has been hindered by the lack of reliable antibodies recognising native N-type calcium channels, which have been validated using knockout tissue. Furthermore, previous studies using antipeptide antibodies have reported conflicting results.^12,29,60,61^ For this reason, we previously developed Ca_V_2.2 constructs containing exofacial epitope tags, in a position not affecting channel function.^9^ We then generated a knockin mouse line carrying the hemagglutinin (HA) tag in this position in the *Cacna1b* gene, to examine the distribution of native Ca_V_2.2 protein in the intact nervous system.^41^ We previously found a dramatic effect of α_2_δ-1 ablation on Ca_V_2.2_HA distribution, with the loss of cell surface Ca_V_2.2, particularly in small peptidergic nociceptive sensory neuron somata and terminals.^41^

Here, we have examined the effect of partial sciatic nerve ligation (PSNL) on Ca_V_2.2_HA distribution in sensory neurons and spinal cord and provide novel insights into cellular pathophysiological mechanisms after nerve injury. We have compared Ca_V_2.2_HA distribution, ipsilateral and contralateral to nerve injury, both in DRG neuronal cell bodies and in their terminals in the dorsal horn, and we have then examined the effect of α_2_δ-1 knockout on this. We have further used several markers of different DRG subtypes, to examine their coexpression with Ca_V_2.2_HA. This includes the glial cell line–derived neurotrophic factor (GDNF) family ligand receptor (GFRα1), which is present in certain low-threshold mechanoreceptors (LTMRs) and is up-regulated following nerve injury.^5,27^ Glial cell line–derived neurotrophic factor is a DRG trophic factor, which is analgesic in neuropathic pain.^7^

Our key finding is that Ca_V_2.2_HA is up-regulated, ipsilateral to PSNL, in medium/large DRG neurons, where it shows increased association with GFRα1. In parallel, we observe increased Ca_V_2.2_HA in ipsilateral medial/central deep dorsal horn, where GFRα1 is correspondingly up-regulated. The increased Ca_V_2.2_HA in DRGs and deep dorsal horn is α_2_δ-1 dependent, whereas the elevation in GFRα1 is not, indicating that it represents increased Ca_V_2.2_HA trafficking to these mechanoreceptor terminals, which may result in elevated neurotransmission.

## 2. Methods

### 2.1. Partial sciatic nerve ligation

The Ca_V_2.2_HA mouse line was generated by Taconic Artemis on the C57BL/6 background, as described in detail previously.^41^ The α_2_δ-1^−/−^ C57BL/6 mouse line described previously^21,44^ was crossed, as heterozygotes, with the Ca_v_2.2_HA knockin mice to generate double-transgenic Ca_v_2.2_HA^KI/KI^ α_2_δ-1^−/−^ mice. Wild-type (WT) mice were C57BL/6. Both male and female mice were used in this study. Mice were housed in groups of no more than 5 on a 12 h: 12 h light: dark cycle; food and water were available ad libitum. All experimental procedures were covered by UK Home Office license, had local ethical approval, and followed the guidelines of the International Association for the Study of Pain.^62^

Surgery was performed based on a method described previously.^44,50^ Mice were maintained under 2% vol/vol isoflurane (Baxter, Northampton, United Kingdom) anesthesia delivered in a 3:2 ratio of nitrous oxide and oxygen. Under aseptic conditions, the left sciatic nerve was exposed through blunt dissection of the biceps femoris above the trifurcation of the nerve. Approximately half of the nerve was ligated with a nonabsorbable 7-0 braided silk thread (Ethicon, VetTech, United Kingdom). The surrounding muscle and skin was closed with absorbable 6-0 vicryl sutures (Ethicon, VetTech) and topical lidocaine cream (5% wt/wt) applied to the skin. Sham surgery was performed in an identical manner, omitting the nerve ligation step. After surgery, the mice were allowed to recover. Foot posture and general behavior of the operated mice were monitored throughout the postoperative period. While blind to genotype, mechanical hypersensitivity was tested 14 days after surgery to confirm that the operated mice used for the study displayed neuropathic responses.

### 2.2. Immunohistochemistry

For immunohistochemistry, on days 14 or 15 after surgery, mice were deeply anaesthetized with an intraperitoneal injection of pentobarbitone (Euthatal, Merial Animal Health, Harlow, United Kingdom; 600 mg/kg), perfused transcardially with saline containing heparin, followed by perfusion with 4% paraformaldehyde in 0.1 M phosphate buffer (PB, pH 7.4) at a flow rate of 2.5 mL·min^−1^ for 4 minutes. Lumbar 4 DRGs and the lumbar enlargement of the spinal cord were dissected out. Following dissection, the spinal cord was postfixed for 2 hours, whereas the DRGs did not undergo extra fixation. Tissue was washed with PB, cryoprotected by incubation in PB with 20% sucrose overnight, and finally mounted in Optimal cutting temperature (OCT) compound (VWR International, Lutterworth, United Kingdom) before storing at −80°C, until sectioning. Dorsal root ganglia and spinal cord were sectioned at 15 and 20 μm, respectively, using a cryostat, placing the sections sequentially in series of 6 slides, so the distance between any section and the next on any slide is 90 or 120 µm in each case. Slides were stored at −80°C until processed.

For immunofluorescence labelling of DRGs, sections were blocked with 10% goat serum in PBS containing 0.3% Triton X-100 for more than 1 hour at room temperature (RT), followed by incubation with the unconjugated goat Fab antimouse IgG (H + L) (0.1 mg/mL in PBS, Jackson ImmunoResearch Lab, Stratech Ltd, Ely, United Kingdom, catalogue number 115-007-003) for 1 hour at RT to reduce nonspecific binding of antirat antibody to endogenous IgG in mouse tissue, washed in PBS, 0.1% Triton X-100 (PBS-T), and then incubated with rat monoclonal anti-HA antibody (Roche, catalogue number 11867423001, 1:100), for 2 to 3 days at 4°C in 5% goat serum, 0.3% Triton X-100 in PBS. Following extensive washing in PBS-T, immunolabelled samples were fixed in 4% paraformaldehyde in PBS for 30 minutes at RT, washed in PBS-T and incubated for 1 to 2 days at 4°C with the goat antirat conjugated with Alexa Fluor 488 (Invitrogen, Thermo Fisher Scientific, Oxford, United Kingdom, catalogue number A11006, 1:500). After washing, sections were treated with the nuclear stain DAPI (Molecular Probes, catalogue number D106, 0.5 μM) and mounted in VectaShield (Vector Laboratories, 2BScientific Ltd., Upper Heyford United Kingdom, catalogue number H-1000). When costaining HA with the goat antibody against GFRα1 (R&D Systems, Bio-techne, Abingdon , United Kingdom, catalogue number AF560, 1:200), the procedure was the same except that horse serum was used instead of goat serum in the blocking and antibody solution, the goat Fab antimouse was omitted, and the secondary antibodies were donkey antigoat conjugated with Alexa Fluor 488, and the donkey antirat highly cross-adsorbed antibody conjugated with Alexa Fluor 594 (Thermo Fisher Scientific, catalogue numbers A11055 and A21209 respectively, both used at 1:500).

For spinal cord immunohistochemistry, sections were incubated with rat monoclonal anti-HA antibody (as above) and costained with rabbit anti-calcitonin gene-related peptide (CGRP), IB4 conjugated with FITC (Sigma, catalogue numbers C8198 and L2895), or with goat anti-GFRα1. Some sections were labelled for α_2_δ-1, as described previously,^44^ with the following modifications: after heat-induced epitope retrieval (10 mM citrate buffer, pH 6.0, 0.05% Tween 20, 95°C for 10 minutes), the sections were washed, blocked with 10% goat serum in PBS containing 0.3% Triton and treated with the unconjugated goat Fab anti-mouse IgG (H + L) (0.1 mg/mL in PBS) for 1 hour at RT. Mouse monoclonal antidihydropyridine receptor (α_2_-1 subunit) antibody (Sigma, D219, 1:100) was applied for 2 or 3 days at 4°C. After extensive washes, the samples were incubated with biotin-conjugated goat antimouse Fab fragment (1:500, Jackson Immuno Research Lab, catalogue number 115-067-003), overnight at 4°C, followed by washes and Streptavidin-AlexaFluor-488 overnight at 4°C (1:500, Invitrogen, catalogue number S32354).

### 2.3. Confocal image acquisition and analysis

Immunostaining was visualized using an LSM 780 (Carl Zeiss UK Ltd., Cambourne, United Kingdom) confocal microscope. Images were acquired with constant settings in each experiment from at least 3 sections per DRG or 7 per spinal cord from at least 3 mice unless otherwise stated. Only intact tissue sections, unfolded and with uniform staining, were selected for imaging and analysis. For DRG sections, multiple images were acquired with a 63× 1.4NA objective (0.7 μm optical section) covering the whole area of the section containing neurons using the tiling mode with a 5% overlap and stitched with Zen software (Zeiss). For analysis, using ImageJ software (Schneider et al., 2012), in every intact DRG neuron with a visible nucleus, we selected 2 different types of regions of interest (ROI). Using images with temporarily enhanced brightness and contrast, solely to aid visualization of the circumference of even dimly stained cells, first we drew a 10 pixel-wide line (0.9 µm) following the perimeter of the cell from which we recorded the length as an estimation of the size of the cell (small <61 µm, medium 61-94 µm, or large >94 µm) and the mean membrane intensity. Next, we selected an ROI for the area inside the first ROI, excluding the plasma membrane and the nucleus, to record the mean intracellular intensity. Regions of interest outside each section were used as background and deducted from sample measurements.

To determine the proportion of DRG neurons expressing Ca_V_2.2_HA, GFRα1 or both, cells with staining above a threshold (3× the SD of the contralateral side from each animal) were selected using the multipoint tool of ImageJ and merged with the list of ROIs around the perimeter of each section, as described above, to quantify the different populations according to staining and size.

Spinal cord images (at least 7 per animal, unless otherwise stated) at low magnification were acquired using a 20× 0.8 NA objective (5-µm optical section) covering the whole dorsal half of each section also in tiling mode and were stitched with Zen software as for DRG sections. For analysis, using the same software, the mean intensity was recorded from a profile scan of a rectangular ROI of 50 × 300 µm placed across the superficial layers of the medial, central, and lateral part of the ipsilateral and contralateral dorsal horn of each section. The ratio of ipsilateral to contralateral per ROI was calculated to determine the relative level of change in fluorescence and then averaged per each animal. To quantify that data, the mean of the superficial (0-80 μm) or deep (140 and 300 μm) regions was extracted. The HA, CGRP, IB4, and GFRα1 data from different experiments were pooled according to genotype and presented as the mean ± SEM.

For high magnification examination of the spinal cord sections, a 63× 1.4 NA objective in Airyscan mode (0.2-µm optical sections) for HA, CGRP, IB4, and GFRα1 was used in tiling mode to generate multiple images covering the ipsilateral or contralateral dorsal horn of each section; superresolution images then underwent Airyscan processing and were stitched using Zen software. To quantify density of Ca_V_2.2_HA immunoreactivity and that of the other markers, a square ROI (70 × 70 µm) in the deeper layers of the medial region or the superficial layers of the medial, central, and lateral dorsal horn was analysed with FIJI software 6. Each ROI was split into 2 or 3 channels (depending on number of markers used) and thresholded (4 × the SD of the deeper ROI or the combined 3 superficial ROIs from the contralateral side from each section) to create a mask per channel with the all the clusters between 110 and 2800 pixels (0.2-5 μm^2^) selected using the particle analyzer command. All the clusters were saved as list of ROIs and used in the original image to record their size and mean intensity. The corresponding masks for HA and the other marker/s were merged, and the overlapping clusters (>1%) were extracted using the plugin Binary Feature Extractor from the BioVoxxel Toolbox (http://www.biovoxxel.de), to obtain the associated clusters. Overall, 126 superficial and 42 deeper ROIs were analysed, from a total of 21 sections with 2 or 3 sections per mouse stained for HA, CGRP and IB4, or HA and GFRα1, from 2 Ca_V_2.2_HA^KI/KI^ -α_2_δ-1^+/+^ and 2 Ca_V_2.2_HA^KI/KI^ -α_2_δ-1^−/−^ mice per experiment.

### 2.4. Statistical analysis

Data were analysed with GraphPad Prism 7 or 9 (GraphPad software, San Diego, CA) or Origin-Pro 2021 (OriginLab Corporation, Northampton, MA). Where error bars are shown they are SEM, “N” refers to number of mice or clusters, unless indicated otherwise. Statistical significance between 2 groups was assessed by Student *t* test or paired *t* test, as stated. Repeated-measures 2-way ANOVA followed by Šídák's multiple comparisons test was used to analyse the number of clusters according to cluster size (or density) and the side of the spinal cord from multiple sections. Details of statistical test results are given in Figure legends and in Supplementary Information (available at http://links.lww.com/PAIN/B762).

## 3. Results

### 3.1. Effect of partial sciatic nerve ligation on distribution of Ca_V_2.2_HA and α_2_δ-1 in dorsal root ganglion neurons in vivo

**Figure 1.**
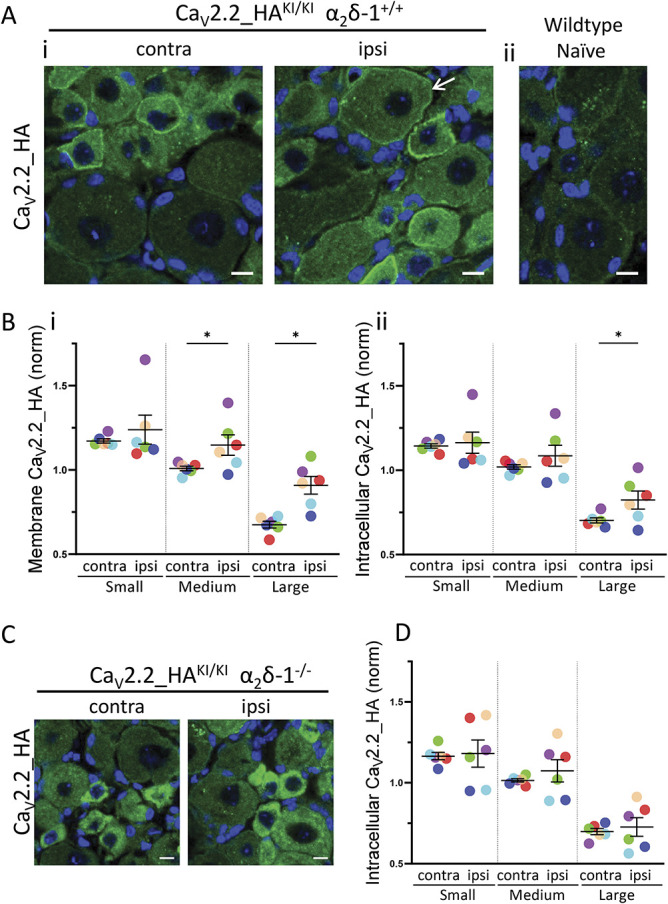
Effect of PSNL and α_2_δ-1 knockout on distribution of Ca_V_2.2_HA in dorsal root ganglia from Ca_V_2.2_HA^KI/KI^ mice. (A) Immunostaining for Ca_V_2.2_HA (green) with nuclear marker DAPI (blue) in DRGs from Ca_V_2.2_HA^KI/KI^, α_2_δ-1^+/+^ mice. (i) Contralateral (contra, left) and ipsilateral (ipsi, right) to PSNL. (ii) Lack of Ca_V_2.2_HA immunoreactivity shown in wild-type naive DRGs. Scale bars are 10 µm. (B) Membrane (i) and intracellular (ii) HA staining for Ca_V_2.2_HA, quantified with respect to cell size (small <61-μm, medium 61- to 94-μm, or large >94-μm perimeter). Data were analysed for 6 mice. For each mouse, all DRG neurons from at least 3 sections from the ipsilateral and contralateral L4 DRG were analysed and normalised to the mean of the contralateral side for each size group (each colour corresponds to mean data from one mouse). Black lines indicate mean ± SEM. Statistical analysis compares ipsilateral and contralateral for each DRG size group, *P < 0.05, paired *t* test. Individual *P* values for small, medium, and large DRGs = 0.4169, 0.0496 and 0.0123 in i; and 0.7651, 0.3030 and 0.0376 in ii, respectively. Data from individual DRGs, and for sham-operated controls are in Supplementary Figs. 1A and B, available at http://links.lww.com/PAIN/B762. (C) Immunostaining for Ca_V_2.2_HA (green) with nuclear marker DAPI (blue) in DRGs from Ca_V_2.2_HA^KI/KI^, α_2_δ-1^−/−^ mice contra (left) and ipsilateral (right) to PSNL. Scale bars are 10 µm. (D) Intracellular HA staining for DRGs from Ca_V_2.2_HA^KI/KI^, α_2_δ-1^−/−^ mice quantified with respect to cell size, from 6 mice, exactly as in (B). Paired *t* test: individual *P* values for small, medium, and large DRGs = 0.8546, 0.4386, and 0.6847, respectively. There was no discernible cell surface staining, and therefore, this was not quantified. Data from individual DRGs are in Supplementary Fig. 1C, available at http://links.lww.com/PAIN/B762. DRG, dorsal root ganglion; HA, hemagglutinin; PSNL, partial sciatic nerve ligation.

The use of Ca_V_2.2_HA knockin mice has revealed that Ca_V_2.2_HA is present both intracellularly and on the cell surface of DRG neuronal cell bodies.^41^ We confirmed this distribution in our current experiments using sections of dorsal root ganglia from 12- to 16-week-old Ca_V_2.2_HA^KI/KI^ mice that have undergone unilateral PSNL (Fig. 1A, i). Immunoreactivity for HA was absent from Ca_V_2.2^WT/WT^ mice (Fig. 1A, ii). In agreement with our previous quantification,^41^ the level of both cell surface and intracellular Ca_V_2.2_HA was highest in small DRG cell bodies (Fig. 1B, i and ii), and it was very low in large DRG neuronal somata contralateral to the PSNL injury (Fig. 1B, i and ii).

Following PSNL, the Ca_V_2.2_HA signal was significantly increased on the cell surface, ipsilateral to the nerve injury, compared with the contralateral side, particularly in large and medium DRG neurons (by 34.6% and 13.8%, respectively) but not in small DRG neurons (Fig. 1A, arrow, Fig. 1B, i). Furthermore, analysis of intracellular Ca_V_2.2_HA showed that although intracellular Ca_V_2.2_HA density was highest in small DRG neurons (Fig. 1A, 1B, ii), it was increased ipsilateral to PSNL relative to the contralateral side, only in large DRG neurons (by 17.1%, Fig. 1B, ii). There was no increase in Ca_V_2.2_HA in sham-operated animals (Supplementary Fig. 1B compared with A, which also shows the data from individual DRG neurons for these experiments, available at http://links.lww.com/PAIN/B762). Together, these results indicate that PSNL produces an increase of Ca_V_2.2_HA in large and medium DRG neurons ipsilateral to the injury, particularly on their cell surface.

### 3.2. Genetic ablation of α_2_δ-1 prevents the increase of Ca_V_2.2_HA in dorsal root ganglion neurons ipsilateral to partial sciatic nerve ligation

It has been found in several studies that α_2_δ-1 is up-regulated following sensory nerve injury and is important for the development of neuropathic allodynia and mechanical hypersensitivity.^4,30,40,44^ We therefore examined the effect of α_2_δ-1 knockout on Ca_V_2.2_HA distribution following PSNL. We observed that the Ca_V_2.2_HA signal at the cell surface of DRG neurons was almost abolished in α_2_δ-1^−/−^ mice (Fig. 1C), as we had found previously.^41^ Following PSNL in these mice, there was no appearance of Ca_V_2.2_HA on the cell surface of the DRG neurons (Fig. 1C, ipsilateral) or any increase in intracellular Ca_V_2.2_HA signal ipsilateral to PSNL (Figs. 1C and D). These results highlight the importance of α_2_δ-1 in the elevation of Ca_V_2.2 in large and medium DRGs that we observed ipsilateral to PSNL.

### 3.3. Ca_V_2.2_HA is decreased in patches of superficial dorsal horn ipsilateral to partial sciatic nerve ligation, in parallel with loss of IB4 and CGRP

**Figure 2.**
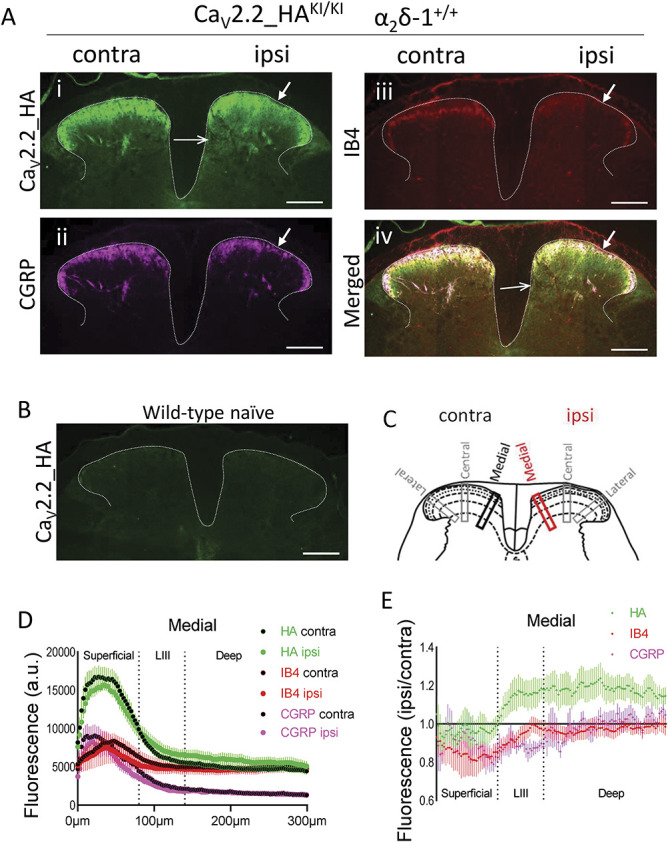
Comparison of the effect of PSNL and α_2_δ-1 knockout on Ca_V_2.2_HA, IB4, and CGRP distribution in superficial dorsal horn. (A) Representative images of dorsal horn sections following PSNL, in Ca_V_2.2_HA^KI/KI^, α_2_δ-1^+/+^ mice. Images are always oriented as contralateral (left) and ipsilateral (right) to PSNL, and stained for Ca_V_2.2_HA (i; green), CGRP (ii; magenta), and IB4 (iii; red). Panel (iv) shows merged images. Solid arrows, areas of decreased staining in superficial dorsal horn. open arrows, area of increased staining of Ca_V_2.2_HA in medial deep dorsal horn. Scale bar: 200 μm. (B) Lack of Ca_V_2.2_HA immunoreactivity in wild-type naive dorsal horn. Scale bar: 200 µm. (C) Diagram of spinal cord dorsal horn showing the position of the 3 ROIs (medial, central, and lateral; 300 × 50 µm) placed in dorsal horn sections, ipsilateral (red) and contralateral (black) to side of PSNL, to measure the fluorescence intensity of Ca_V_2.2_HA and other markers. (D) Immunofluorescence profiles for Ca_V_2.2_HA (green), IB4 (red), and CGRP (magenta) in the medial ROI shown in (C), contralateral (black-filled symbols) and ipsilateral (color-filled symbols) to PSNL in Ca_V_2.2_HA^KI/KI^, α_2_δ-1^+/+^ dorsal horn. Data are the mean ± SEM for N = 5 mice (7 sections/mouse). (E) Ratio ROI profiles (ipsilateral/contralateral) for data shown in D, for Ca_V_2.2_HA (green), IB4 (red), and CGRP (magenta). Data are the mean ± SEM for N = 5 mice (7 sections/mouse). Quantification of mean average of superficial and deep laminae for CGRP and IB4 from this data and from Ca_V_2.2_HA^KI/KI^, α_2_δ-1^−/−^ are shown in Supplementary Fig. 2, available at http://links.lww.com/PAIN/B762. HA, hemagglutinin; PSNL, partial sciatic nerve ligation; ROI, regions of interest.

Next, we examined the effect of PSNL on the distribution of Ca_V_2.2_HA, in the dorsal horn of the spinal cord, in parallel with other DRG subtype markers, CGRP and IB4 (Fig. 2). As we previously described,^41^ there is strong immunoreactivity for Ca_V_2.2_HA in the superficial laminae I and II of the dorsal horn (Fig. 2A, i), and there was no signal in naive WT mice (Fig. 2B). This localization shares topographic distribution with the presynaptic marker CGRP, which is present in peptidergic nonmyelinated primary afferent C-fiber terminals in laminae I and II-outer (Fig. 2A, ii), and also with IB4, which is present in nonpeptidergic primary afferent C-fiber terminals, mainly in lamina II-inner (Fig. 2A, iii).

Following PSNL, we observed a patchy loss of staining for IB4, and to a lesser extent CGRP, ipsilateral to the nerve injury, which was paralleled by a loss of Ca_V_2.2_HA (Fig. 2A, ipsilateral on right side of each section, i-iii, and merged image in iv; closed arrows). This irregular loss of IB4 and CGRP staining has previously been described in many studies, and it is believed to be due to deafferentation of neurotrophin-dependent terminals following nerve injury.^2,38,55^ For the first time, we can now see that Ca_V_2.2_HA present in those terminals is also reduced. To quantify the observed signals, and the effect of PSNL, we took ROIs perpendicular to the pial surface in medial, central, and lateral regions of the dorsal horn (Fig. 2C) and quantified the fluorescence intensity profiles through the different laminae (Fig. 2D, data shown for the medial ROI), as described previously.^41^ We then determined the ratio of ROI intensity (ipsilateral/contralateral for each section) with respect to the PSNL injury (Fig. 2E). For both IB4 and CGRP, taking the mean intensity for each animal for the superficial layers (Supplementary Fig. 2B, C, available at http://links.lww.com/PAIN/B762), although there is an obvious patchy reduction in most cases, no significant overall reduction was observed, presumably because of the irregular and variable nature of the loss in this nerve injury model. The Ca_V_2.2_HA signal showed a similar patchy loss of staining in the same areas as found for IB4 and CGRP (Fig. 2A, iv, solid arrow in merged image), suggesting that it may be present in the same terminals, as we previously concluded using dorsal rhizotomy and high-resolution microscopy.^41^

### 3.4. Superresolution analysis of distribution of Ca_V_2.2-HA in superficial dorsal horn following partial sciatic nerve ligation: comparison with distribution of IB4 and CGRP

**Figure 3.**
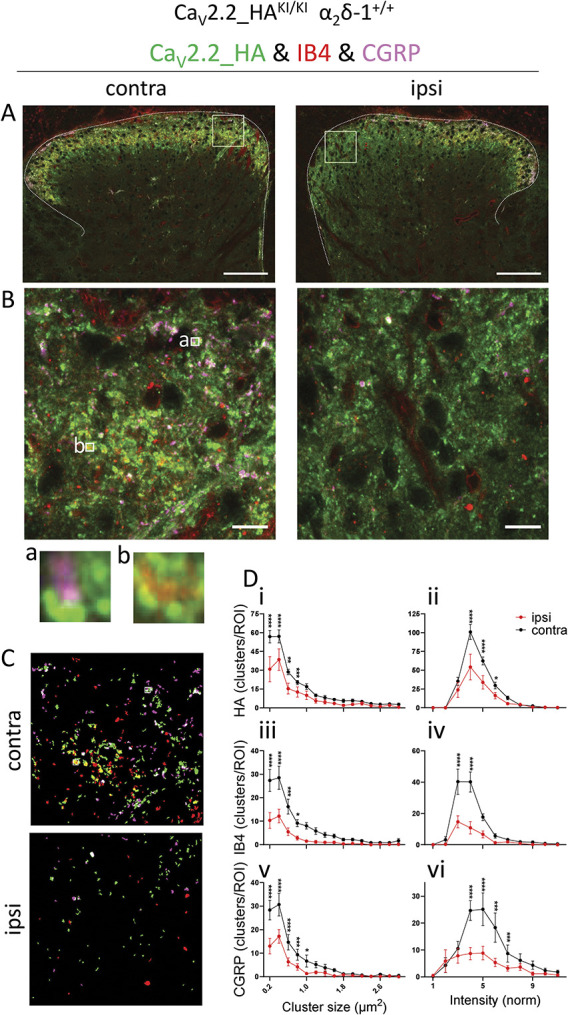
High-resolution analysis of Ca_V_2.2_HA, IB4, and CGRP clusters in superficial dorsal horn following PSNL. (A and B) Representative Airyscan tiled images of contralateral (left) and ipsilateral (right) dorsal horn from the same section following PSNL stained for Ca_V_2.2_HA (green), IB4 (red), and CGRP (magenta). ROIs (70 × 70 μm) were placed in the medial, central, and lateral regions of the superficial layer for quantification of clusters. The medial ROI is indicated with a square in (A) and shown enlarged in (B). Representative CGRP (a) and IB4 (b) positive glomeruli are indicated by small ROIs (2 × 2 μm) in the contralateral side of (B) and enlarged underneath. Scale bars in (A and B): 100 μm and 10 μm, respectively. (C) Composite mask from the 3 channels (Ca_V_2.2_HA [green], IB4 [red], and CGRP [magenta]) from ROIs as shown in (B), for particles between 0.2 and 5 μm^2^, with signal above threshold (see Methods). (D) Size and intensity distribution of clusters positive for Ca_V_2.2_HA, IB4, and CGRP on the ipsilateral (red) and contralateral (black) sides in the medial superficial ROI. N =545, 126, and 166 (ipsi) and 1058, 500, and 470 (contra) clusters for Ca_V_2.2_HA-, IB4-, and CGRP-positive clusters, respectively. Data from 10 superficial medial ROIs (5 contra and 5 ipsi, from 2 or 3 sections per each of 2 mice). Statistical significances are given by *P < 0.05, **P < 0.01, ***P < 0.001, ****P < 0.0001. Details of statistical test results (Repeated-measures 2-way ANOVA followed by Šídák's multiple comparisons test) are in Supplementary Information, available at http://links.lww.com/PAIN/B762. ANOVA, analysis of variance; CGRP; calcitonin gene-related peptide; HA, hemagglutinin; PSNL, partial sciatic nerve ligation; ROI, regions of interest.

We then analysed superresolution Airyscan images taken from ROIs from the medial, central, and lateral regions of the superficial dorsal horn sections contralateral and ipsilateral to PSNL (ROI for medial region shown in Figs. 3A and B) and examined the size and intensity of Ca_V_2.2_HA, CGRP, and IB4 clusters and their association (mask and data for medial ROI shown in Figs. 3C and D). Ca_V_2.2_HA, together with either IB4 or CGRP, are found in rosette-like glomerular clusters in the superficial dorsal horn (images A and B, below Fig. 3B), as previously observed.^41^ Quantification of all the clusters from the combined ROIs contralateral to PSNL in superficial dorsal horn shows that 35.2% of IB4 and 34.1% of CGRP clusters were associated with Ca_V_2.2_HA. Similarly, 22.5% and 19.1% of Ca_V_2.2_HA clusters were associated with IB4 and CGRP, respectively (data determined from 15 ROIs, 3 from each side, in 5 sections from 2 mice; 2607 Ca_V_2.2 clusters, 1657 IB4 clusters, and 1442 CGRP clusters). We also examined the effect of PSNL on the area and intensity of Ca_V_2.2_HA, CGRP, and IB4 clusters with respect to their density of distribution. We found that only the density of Ca_V_2.2_HA clusters (number of clusters/ROI) was decreased ipsilateral to nerve injury (Figs. 3C and 3D, i-ii), with no clear change in size profile (Fig. 3D, i) or intensity distribution (Fig. 3D, ii). Similarly, the density of both IB4 clusters (Fig. 3D, iii-iv) and CGRP clusters (Fig. 3D, v-vi) decreased ipsilateral to PSNL, again with no change in size profile (Fig. 3D, iii, v) or intensity distribution (Fig. 3D, iv, vi).

Taken together, these results show that the patchy reduction observed at low magnifications for Ca_V_2.2_HA, IB4, and CGRP in the superficial dorsal horn, ipsilateral to PSNL, corresponds to a decreased number of glomerular clusters rather than a change in their size or intensity in response to the nerve injury.

### 3.5. Effect of α_2_δ-1 knockout on the Ca_V_2.2_HA distribution in the superficial dorsal horn

**Figure 4.**
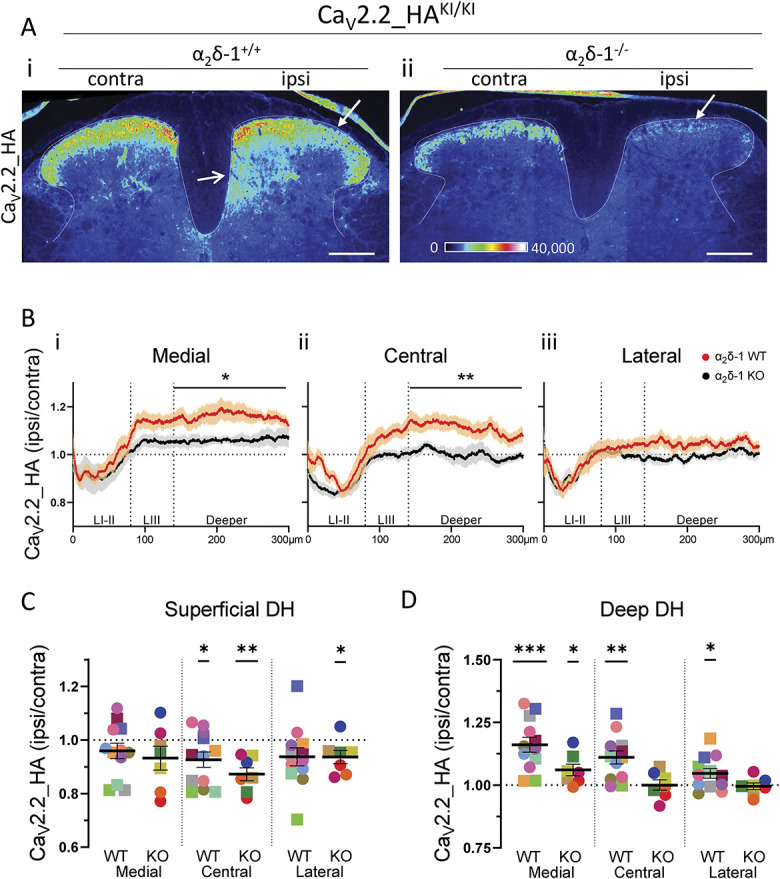
Quantification of the effect of α_2_δ-1 knockout on distribution of Ca_V_2.2_HA in the superficial and deep dorsal horn of Ca_V_2.2_HA^KI/KI^ mice following PSNL. (A) Representative images for Ca_V_2.2_HA immunostaining (depicted as Rainbow look-up table, LUT), from Ca_V_2.2_HA^KI/KI^, α_2_δ-1^+/+^ (i) and Ca_V_2.2_HA^KI/KI^, α_2_δ-1^−/−^ (ii) mice following PSNL. Sections are oriented as contra (left) and ipsi (right) to PSNL. Closed arrows indicate patchy loss of Ca_V_2.2_HA in superficial dorsal horn, and open arrow in (i) indicates increase in Ca_V_2.2_HA in deep dorsal horn, ipsilateral to PSNL. Scale bars 200 μm. (B) Plots of the change in Ca_V_2.2_HA intensity following PSNL (expressed as a ratio of ipsi to contra) in the medial (i), central (ii), and lateral (iii) ROIs from Ca_V_2.2_HA^KI/KI^, α_2_δ-1^+/+^ (α_2_δ-1 WT, N = 12 mice, red) and Ca_V_2.2_HA^KI/KI^, α_2_δ-1^−/−^ (α_2_δ-1 KO, N = 7 mice, black). A total of 7 sections were averaged per mouse. Data are mean ± SEM of the number of mice stated. Statistical significance between α_2_δ-1^+/+^ and α_2_δ-1^−/−^ in the region indicated was determined by unpaired *t* test (*P < 0.05, **P < 0.01). The individual *P* values for LI-II, LIII and deeper layers are, respectively, 0.6077, 0.0611, and 0.0331 in (i); 0.2115, 0.0864, and 0.0096 in (ii): and 0.9912, 0.4349, and 0.0748 in (iii). (C, D) Quantification of the change in immunofluorescence of Ca_V_2.2_HA (expressed as a ratio of ipsi/contra) in (C) superficial layer (laminae I and II, from pial surface to 80 μm) and (D) deeper layers (laminae IV and part of V, from 140 to 300 μm) of the dorsal horn. Data are given for α_2_δ-1^+/+^ mice (WT, N = 12) or α_2_δ-1^−/−^ mice (KO, N = 7). Each coloured symbol represents the mean ratio for each animal (squares represent male and circles female mice), and the black line represents mean ± SEM. Statistical significance of ipsilateral/contralateral data determined by one sample *t* test with 1 as reference value (*P < 0.05, **P < 0.01, ***P < 0.001). The individual *P* values for medial, central, and lateral regions are 0.1870, 0.0261, and 0.0883 for α_2_δ-1^+/+^ and 0.1786, 0.0019, and 0.0396 for α_2_δ-1^−/−^, respectively in (C) and 0.0002, 0.0014, and 0.0309 for α_2_δ-1^+/+^ and 0.0391, 0.9829, and 0.7527 for α_2_δ-1^−/−^ in (D), respectively. DH, dorsal horn; HA, hemagglutinin; PSNL, partial sciatic nerve ligation; ROI, regions of interest.

We next examined the effect of genetic ablation of α_2_δ-1 on the changes in Ca_V_2.2_HA distribution in the dorsal horn following PSNL. We have previously shown that the signal for Ca_V_2.2_HA in the dorsal horn was markedly reduced in Ca_V_2.2_HA^KI/KI^/α_2_δ-1^−/−^ mice, particularly in the superficial laminae,^41^ and this is confirmed here (Fig. 4A ii, compared with i). However, we found here that the patchy reduction in Ca_V_2.2_HA following PSNL (Fig. 4A, i) in the superficial layers of the dorsal horn was still evident in α_2_δ-1^−/−^ mice (Fig. 4A, ii). We further quantified the PSNL-mediated reduction of Ca_V_2.2_HA in laminae I and II of the dorsal horn, to examine the effect of α_2_δ-1^-^ knockout (KO), by means of the ROI ipsilateral:contralateral ratio profiles (Fig. 4B, α_2_δ-1^+/+^ [red] compared with α_2_δ-1^−/−^ [black] intensity profiles), which show that the reduction in laminae I-II remains evident in all ROIs of α_2_δ-1^−/−^ dorsal horn (Fig. 4B, i-iii). This is confirmed from the mean intensity ratios for laminae I-II, which showed a statistically significant reduction in the central and lateral ROIs of α_2_δ-1^−/−^ mice (Fig. 4C). There were no evident differences between the results in male and female mice for either genotype (Fig. 4C and Supplementary Table 1, available at http://links.lww.com/PAIN/B762).

In parallel, the patchy reduction in IB4 and CGRP signal, ipsilateral to PSNL injury in the superficial ROIs also remained evident in α_2_δ-1^−/−^ dorsal horn (Supplementary Fig, 2A, vi-vii, arrows, available at http://links.lww.com/PAIN/B762), although it was only statistically significant for IB4 (Supplementary Fig. 2B, C, available at http://links.lww.com/PAIN/B762), indicating that these PSNL-induced reductions were not α_2_δ-1 dependent. In Airyscan analysis of sections from α_2_δ-1^−/−^ dorsal horn (Supplementary Fig. 3A-D, available at http://links.lww.com/PAIN/B762), we found the reductions in Ca_V_2.2_HA, IB4, and CGRP cluster density ipsilateral to PSNL were still present in superficial dorsal horn (Supplementary Fig. 3D i-vi, available at http://links.lww.com/PAIN/B762), with no change in size (Supplementary Fig. 3D i, iii, v, available at http://links.lww.com/PAIN/B762) or intensity distribution (Supplementary Fig. 3D ii, iv, vi, available at http://links.lww.com/PAIN/B762).

Together, these results show that Ca_V_2.2_HA is present in the glomerular nerve terminals in the superficial dorsal horn that undergo nerve injury–dependent deafferentation ipsilateral to PSNL, and this partial loss is not α_2_δ-1 dependent.

### 3.6. Ca_V_2.2_HA is increased in deep dorsal horn of spinal cord ipsilateral to partial sciatic nerve ligation

Surprisingly, we observed a consistent and marked increase in Ca_V_2.2_HA ipsilateral to PSNL, in the deep layers of the dorsal horn (layers IV-V, Fig. 2A, i; Fig. 4A, i, open arrows), particularly in the medial and central ROIs (Fig. 2E green profile; Fig. 4B, orange profiles, i, ii), which was less evident in the lateral ROI (Fig. 4B, iii). Quantification shows a statistically significant increase in the deep dorsal horn for all 3 regions (Fig. 4D). There were no evident differences between the results in male and female mice for either genotype (Fig. 4D and Supplementary Table 1, available at http://links.lww.com/PAIN/B762). By contrast, there was no parallel increase in CGRP or IB4 in the deep dorsal horn, indicating that the increase was not due to sprouting of these terminals into the deeper layers (Fig. 2A, ii-iv; Fig. 2E).

### 3.7. Ablation of α_2_δ-1 almost abolishes the partial sciatic nerve ligation–induced increase in Ca_V_2.2_HA in deep dorsal horn

Ablation of α_2_δ-1 almost abolished the effect of PSNL on the increase of Ca_V_2.2_HA in the deep dorsal horn (laminae IV-V) (Fig. 4A, ii). The ROI ipsilateral:contralateral ratio profiles show that the PSNL-induced increase in Ca_V_2.2_HA distribution in the ipsilateral medial and central deep dorsal horn was lost or markedly reduced in α_2_δ-1^−/−^ mice (Fig. 4B, black intensity profiles), in contrast to the increase observed ipsilateral to PSNL in α_2_δ-1^+/+^ deep dorsal horn (Fig. 4B, orange intensity profiles). Quantification confirmed that the PSNL-mediated increase is lost in the central and lateral ROIs and much reduced in the medial ROI (Fig. 4D). Together, these results indicate that the presence of α_2_δ-1 (which is elevated following PSNL, see next section) is key to the increase in Ca_V_2.2_HA in the medial and central deep dorsal horn ipsilateral to PSNL.

### 3.8. Expression of α_2_δ-1 is increased ipsilateral to partial sciatic nerve ligation in deep dorsal horn

**Figure 5.**
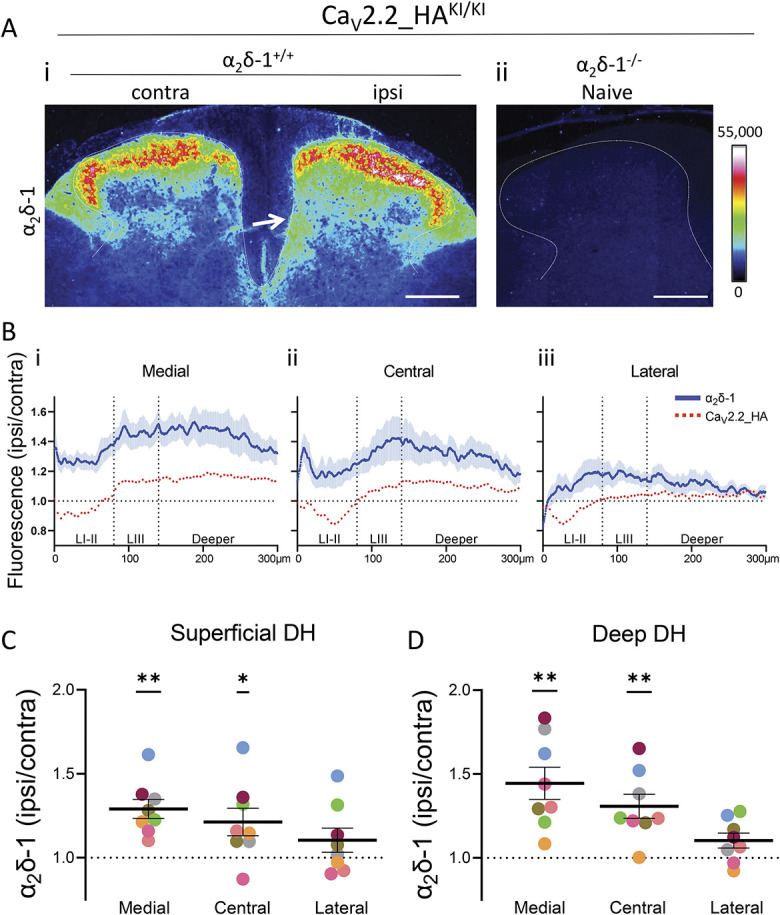
Distribution of α_2_δ-1 in the dorsal horn of Ca_V_2.2_HA knockin mice following PSNL. (A) Representative image for α_2_δ-1 immunostaining from Ca_V_2.2_HA^KI/KI^ mice for α_2_δ-1^+/+^(i) following PSNL, and naive α_2_δ-1^−/−^ (ii) mice. Open arrow in (i) indicates increase of α_2_δ-1 in (Rainbow LUT) in deep dorsal horn, ipsilateral to PSNL. Scale bar 200 μm. (B) Plots of the PSNL-induced change in intensity for Ca_V_2.2_HA (orange dotted lines, same data as in Fig. 3B, for ease of comparison) and α_2_δ-1 (blue lines) immunostaining (expressed as a ratio of ipsilateral/contralateral ROI profiles), in the medial (i), central (ii), and lateral (iii) ROIs (mean ± SEM for N = 12 and 8 mice for Ca_V_2.2_HA and α_2_δ-1 immunostaining, respectively). A total of 7 sections were analysed per mouse in each condition, except for 2 mice stained for α_2_δ-1 where only 4 or 6 sections were available for analysis (mean ± SEM). (C, D) Quantification of the change in α_2_δ-1 immunofluorescence (ipsilateral/contralateral) in Ca_V_2.2_HA^KI/KI^ mice for (C) superficial layer (laminae I and II, from pial surface to 80 μm) and (D) deeper layers (laminae IV and V, from 140 to 300 μm) for the medial, central, and lateral ROIs. N = 8 mice, with each symbol representing the mean for each animal, and black line represents mean ± SEM. Statistical significance determined by one sample *t* test with 1 as reference value (*P < 0.05, **P < 0.01). The individual *P* values for medial, central, and lateral regions are 0.0013, 0.0358, and 0.1890, respectively in (C); and 0.0024, 0.0036, and 0.0534, respectively in (D). DH, dorsal horn; HA, hemagglutinin; PSNL, partial sciatic nerve ligation; ROI, regions of interest.

In parallel experiments, immunostaining for α_2_δ-1 confirmed its strong expression in the superficial layers of the dorsal horn and its up-regulation ipsilateral to PSNL injury (Fig. 5A, i). The specificity of staining is confirmed by its absence in α_2_δ-1^−/−^ dorsal horn (Fig. 5A, ii). Interestingly, α_2_δ-1 up-regulation shows a minimum in layers I and II from the intensity profiles (Fig. 5B, i and ii, blue profiles), in parallel with the patchy reduction in Ca_V_2.2_HA signal, particularly in the medial and central ROIs (Fig. 5B, blue compared with the dotted orange intensity profiles, repeated from Fig. 4B for comparison). In addition to the increase of α_2_δ-1 in the superficial layers (Fig. 5C), there is also a marked increase of the α_2_δ-1 signal in layers IV-V of the deep dorsal horn ipsilateral to PSNL (Fig. 5A, open arrow; Fig. 5B, blue intensity profiles), which is evident in the medial and central ROIs (Fig. 5B, i-ii; Fig. 5D).

These data show that α_2_δ-1 is extensively increased in the ipsilateral dorsal horn following PSNL in mice, as previous studies have demonstrated for other nerve injury models. Here, we also note that there is a differential up-regulation according to the laminae, with a lower increase in the superficial layers and greater relative up-regulation in the deeper layers, particularly in the central and medial regions, in parallel with the changes observed for Ca_V_2.2_HA.

### 3.9. Elevation of Ca_V_2.2-HA in deep dorsal horn following partial sciatic nerve ligation parallels an increase of GFRα1

**Figure 6.**
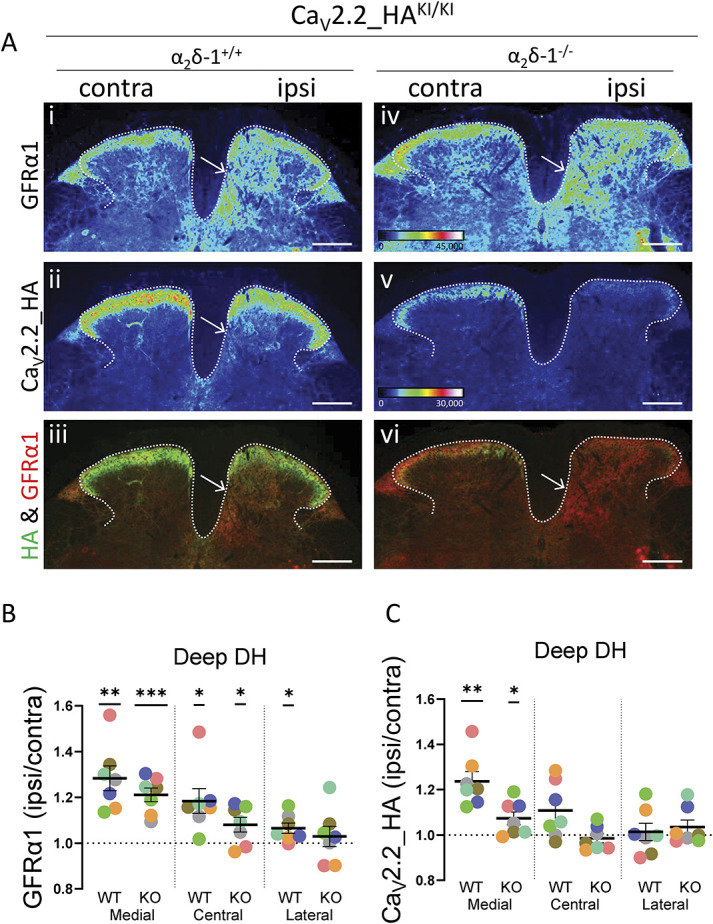
Comparison of distribution of GFRα1 and Ca_V_2.2_HA following PSNL in the deep dorsal horn of Ca_V_2.2_HA^KI/KI^ mice. (A) Representative images of dorsal horn from spinal cord sections following PSNL (Rainbow LUT). Sections from Ca_V_2.2_HA^KI/KI^ mice, either α_2_δ-1^+/+^ (i-iii) or α_2_δ-1^−/−^ (iv-vi) were stained for GFRα1 (top panel, i, iv) and Ca_V_2.2_HA (middle panel, ii, v). Merged images (iii, vi) are shown in bottom panel. Open arrow: area of increased staining in medial deep dorsal horn. Scale bar 200 μm. (B and C) Quantification of the change of GFRα1 (B) and Ca_V_2.2_HA (C) immunostaining following PSNL (expressed as a ratio ipsilateral/contralateral to PSNL) in deeper layers of dorsal horn (Laminae IV and V, from 140 to 300 μm) in the medial, central, and lateral ROIs. Each symbol represents mean data for each mouse (7 sections per mouse). The black bars represent mean ± SEM for N= 7 mice. Statistical difference was determined by one sample *t* test with 1 as reference value. *P < 0.05, **P < 0.01, ***P < 0.001. The individual *P* values for medial, central, and lateral regions are 0.0020, 0.0149, and 0.0265 for α_2_δ-1^+/+^ and 0.0004, 0.0461, and 0.5330 for α_2_δ-1^−/−^, respectively in (B): and 0.0015, 0.0599 and 0.7378 for α_2_δ-1^+/+^; and 0.0411, 0.4833 and 0.3053 for α_2_δ-1^−/−^, respectively in (C). DH, dorsal horn; HA, hemagglutinin; PSNL, partial sciatic nerve ligation; ROI, regions of interest.

Next, we focussed specifically on the medial deep layers of the dorsal horn, in which a strong increase in Ca_V_2.2_HA was observed ipsilateral to PSNL. It has been found previously that the GDNF family receptor α (GFRα1, α2 and α3) proteins that bind GDNF, neurturin, and artemin, respectively, are present in DRG neurons^56^ and are differentially regulated in DRGs following nerve injury.^5,27^ Of particular note, GFRα1 expression is increased in large DRG neurons,^5^ with a corresponding widespread elevation in the dorsal horn,^27^ whereas the same pattern is not shown for GFRα2 and GFRα3.^27^ Therefore, we examined whether there was an increase in GFRα1 immunoreactivity ipsilateral to PSNL, in parallel with the increase in Ca_V_2.2_HA (Fig. 6A). We found that GFRα1 was elevated particularly in the medial and central deep dorsal horn (Fig. 6A, i, arrow; Fig. 6B), and furthermore this increase remained present in α_2_δ-1^−/−^ mice (Fig. 6A, iv; Fig. 6B). In parallel, these experiments confirmed the increase in Ca_V_2.2_HA in the medial deep dorsal horn following PSNL in α_2_δ-1^+/+^ mice (Fig. 6A, ii, arrow; Fig. 6C) and the loss of this effect in α_2_δ-1^−/−^ mice (Fig.6A, v; Fig. 6C).

### 3.10. Increased co-expression of Ca_V_2.2-HA and GFRα1 in dorsal root ganglion neurons following partial sciatic nerve ligation

**Figure 7.**
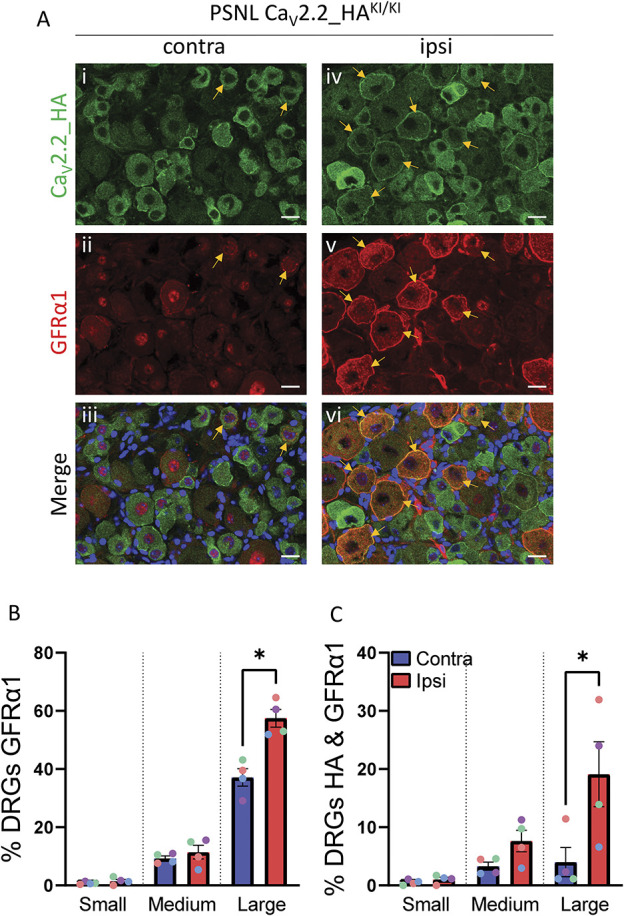
Increased co-localisation of Ca_V_2.2_HA and GFRα1 ipsilateral to PSNL in DRGs from Ca_V_2.2_HA^KI/KI^ mice. (A) Immunostaining for Ca_V_2.2_HA (green, i and iv), GFRα1 (red, ii and v), and merged with DAPI (blue, iii and vi), in contralateral (contra: i, ii and iii) and ipsilateral (ipsi: iv, v and vi) DRG neurons from Ca_V_2.2_HA^KI/KI^ mice following PSNL. Yellow arrows point to neurons coexpressing Ca_V_2.2_HA and GFRα1. Scale bar 20 μm. (B) Percentage of small, medium, and large DRG neurons, contralateral (blue bars) and ipsilateral (red bars) to PSNL, with immunoreactivity above the threshold for GFRα1. Mean ± SEM from 4 mice (different colour symbols represent data from individual mice). Statistical differences were determined by paired *t* test within each size group. *P < 0.05. The individual *P* values for small, medium, and large DRG groups are 0.298, 0.301, and 0.025, respectively. (C) Percentage of small, medium, and large DRG neurons, contralateral (blue bars) and ipsilateral (red bars) to PSNL, with immunoreactivity above the threshold for both Ca_V_2.2_HA and GFRα1. Mean ± SEM from 4 mice (different colour symbols represent data from individual mice). Statistical differences were determined by paired *t* test within each size group. *P < 0.05. The individual *P* values for small, medium, and large DRG groups are 0.336, 0.100, and 0.028, respectively. DRG, dorsal root ganglion; HA, hemagglutinin; PSNL, partial sciatic nerve ligation.

Having identified an increased co-expression of GFRα1 with Ca_V_2.2_HA in the deep dorsal horn following PSNL, we then examined their expression in individual DRG neurons. An increase in immunoreactivity for GFRα1 was previously found in large-diameter DRG neurons in a different nerve injury model.^5^ Here, we found that following PSNL, the proportion of large DRG neurons immunopositive for GFRα1 was increased from 37% on the contralateral side to 57% on the ipsilateral side (Fig. 7A, ii, v; Fig. 7B), whereas there was no significant increase in medium or small DRG neurons (Fig. 7B). Unlike the increase of Ca_V_2.2_HA in large DRG neurons (Fig. 7A, i, iv), the elevated GFRα1 level in DRG neurons following PSNL was not dependent on the presence of α_2_δ-1 (Supplementary Fig. 4, available at http://links.lww.com/PAIN/B762).

We further found that the proportion of DRG neurons that coexpressed Ca_V_2.2_HA and GFRα1 was significantly increased ipsilateral to PSNL only for large DRG neurons, from 4% on the contralateral side to 19% on the ipsilateral side (Fig. 7A, iii, vi; Fig. 7C).

### 3.11. Superresolution analysis of distribution of Ca_V_2.2-HA in deep dorsal horn following partial sciatic nerve ligation: comparison with distribution of GFRα1

**Figure 8.**
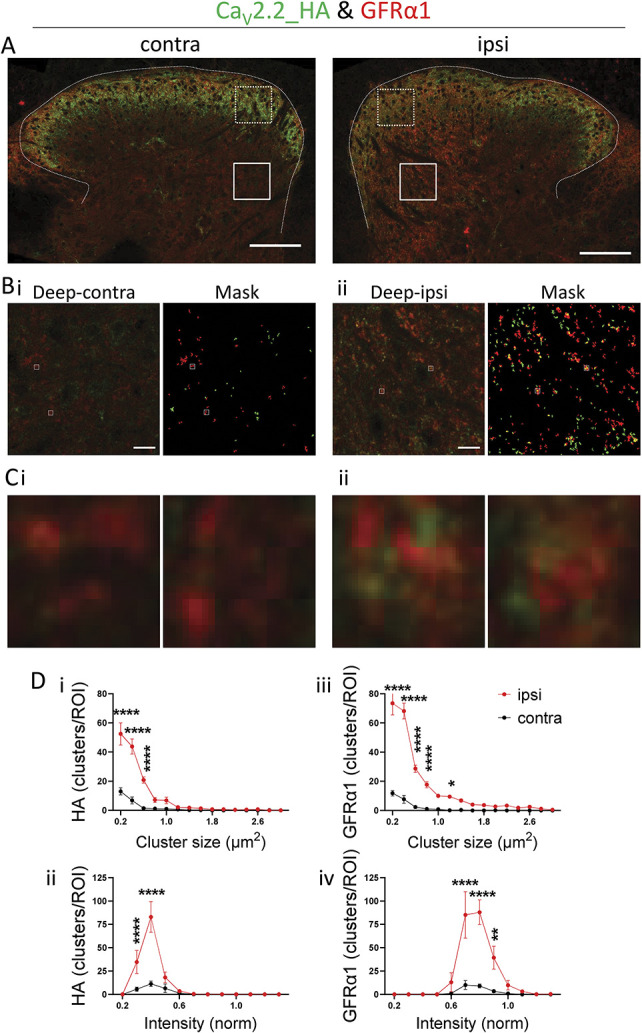
High-resolution analysis of Ca_V_2.2_HA, and GFRα1 clusters in deep dorsal horn following PSNL. (A) Representative Airyscan tiled images of contralateral (left) and ipsilateral (right) dorsal horn from the same section following PSNL, stained for Ca_V_2.2_HA (green) and GFRα1 (red). ROIs (70 × 70 μm) are indicated in the medial superficial (dotted lines) and deep (solid lines) layers of each side, used for cluster analysis. Scale bars 100 μm. (B) Enlarged deep medial contralateral (i) and ipsilateral (ii) ROIs (solid lines from A). Corresponding composite mask from the 2 channels, used for cluster analysis with Ca_V_2.2_HA (green) and GFRα1 (red) are shown on the right of each ROI, with signal above threshold and particles between 0.2 and 5 μm^2^ (see Methods). Scale bars 10 μm. (superficial medial ROIs are shown in Supplementary Fig. 6A, available at http://links.lww.com/PAIN/B762). (C) Enlarged contralateral (i) and ipsilateral (ii) ROIs (2 × 2 μm) as indicated in (B) showing Ca_V_2.2_HA (green) or GFRα1 (red) positive clusters. (D) Size (i, iii) and intensity (ii, iv) distributions of clusters positive for Ca_V_2.2_HA (i, ii) and GFRα1 (iii, iv) for ipsilateral (red) and contralateral (black) medial deep ROIs. N (clusters) = 840 Ca_V_2.2_HA and 1441 GFRα1 (ipsilateral) and 150 Ca_V_2.2_HA and 147 GFRα1 (contralateral). Data are for 6 ROIs from each side, from 3 sections per each of 2 mice. Statistical significances are denoted by *P < 0.05, **P < 0.01, ***P < 0.001, ****P < 0.0001. Details of statistical test results (repeated-measures 2-way ANOVA followed by Šídák's multiple comparisons test) are in Supplementary Information, available at http://links.lww.com/PAIN/B762. ANOVA, analysis of variance; HA, hemagglutinin; PSNL, partial sciatic nerve ligation; ROI, regions of interest.

Because there was a 4.75-fold increase in the coexpression of Ca_V_2.2_HA and GFRα1 in individual large DRG neurons, we next examined whether there was also an increase in the coexpression in their terminal field in the deep dorsal horn. Therefore, we analyzed the association between GFRα1 and Ca_V_2.2_HA in Airyscan images taken from dorsal horn sections contralateral and ipsilateral to PSNL (Fig. 8A). In the medial deep dorsal horn, we found a strong association between Ca_V_2.2_HA and GFRα1 ipsilateral to PSNL (Fig. 8B, ii compared with i). This was clearly evident in the enlarged clusters (Fig. 8C ii compared with i). Analysis showed that 48.8% of Ca_V_2.2_HA clusters were associated with GFRα1, and 28.0% of GFRα1 clusters were associated with Ca_V_2.2_HA ipsilateral to PSNL (Fig. 8C, ii), whereas there was very little observed association on the contralateral side (Fig. 8C, i). Expanding on the low-resolution results in Figure 6, we find that the density of Ca_V_2.2_HA clusters was increased in the deep dorsal horn, ipsilateral to PSNL (Fig. 8D, i and ii), with no change in size profile (Fig. 8D, i) or intensity distribution (Fig. 8D, ii). Further analysis of all combined data for Ca_V_2.2_HA clusters in deep dorsal horn confirmed these conclusions (Supplementary Fig. 5, available at http://links.lww.com/PAIN/B762). In parallel, there was an increase in density of GFRα1 clusters (Fig. 8D, iii-iv), again with no marked change in size profile (Fig. 8D, iii) or intensity distribution (Fig. 8D, iv). We observed no evidence for increased expression of Ca_V_2.2_HA within cell bodies in the dorsal horn following nerve injury.

By contrast, in the superficial dorsal horn, we found that there was little association between GFRα1 and Ca_V_2.2_HA, and this was not increased following PSNL (detailed in Supplementary Fig. 6A-C, available at http://links.lww.com/PAIN/B762), in agreement with their lack of coexpression in small DRG neuronal cell bodies (Fig. 7). Ipsilateral to PSNL, there was a marked reduction of Ca_V_2.2_HA clusters, in agreement with data in Figure 3. By contrast, the density of GFRα1 clusters was increased in the superficial dorsal horn, ipsilateral to PSNL (Supplementary Fig. 6C, available at http://links.lww.com/PAIN/B762).

In parallel analysis of sections from α_2_δ-1^−/−^ dorsal horn (Supplementary Fig. 7A-E, available at http://links.lww.com/PAIN/B762), the increase in GFRα1 cluster density remained present in the deep dorsal horn (Supplementary Fig. 7E, iii-iv, available at http://links.lww.com/PAIN/B762), in agreement with Figure 6. By contrast, in the same ROIs, there was no increase in Ca_V_2.2_HA cluster density in the medial deep dorsal horn, ipsilateral to PSNL (Supplementary Fig. 7E, i-ii, available at http://links.lww.com/PAIN/B762).

Together, these results show a close association between the increase in both Ca_V_2.2_HA and GFRα1 in the medial deep dorsal horn ipsilateral to PSNL. However, because the increase in Ca_V_2.2_HA (but not GFRα1) is α_2_δ-1 dependent, it likely involves an increase in Ca_V_2.2_HA trafficking into terminal fields in which GFRα1 is also elevated.

## 4. Discussion

Ca_V_2.2 channels play an essential presynaptic role in neurotransmitter release in primary afferent terminals^8,11^ and are a therapeutic target in treatment of neuropathic pain. Indeed, N-type Ca_V_ channel blockers alleviate chronic pain in both animal models and humans.^37,43,51^ However, until now, it has not been possible to examine accurately the distribution of endogenous N-type channels, and the effect of an animal model of chronic pain, on tissue expression and distribution of the relevant endogenous N-type channels.

In this study, we have examined the effect of a chronic neuropathic pain model (PSNL) on the distribution of native N-type Ca_V_2.2 channels in DRGs and dorsal horn of the spinal cord, using a knockin mouse, in which Ca_V_2.2 contains an HA epitope tag to aid its identification. This tag does not affect channel function.^9^ In our previous study using these mice, we showed that Ca_V_2.2_HA is strongly expressed on the cell surface of DRG neurons, particularly of the small CGRP-positive nociceptors, and in parallel, there is strong expression in the dorsal horn of the spinal cord, mainly in laminae I and II, corresponding to the primary afferent C-nociceptor glomerular terminals.^41^

Ca_V_2.2 channels have been estimated to comprise 20 to 50% of the total calcium current in DRG somata, depending on species, developmental stage, culture conditions, and DRG neuron subtype.^15,39,44,48,49^ For example, Murali et al.^39^ found 40% N-type calcium current in small DRGs and 20% in larger DRGs. Despite the proviso that DRG neurons placed in culture may undergo rapid changes in ion channel cell surface expression,^19^ nevertheless, we found greater expression of Ca_V_2.2_HA in small and medium, relative to large DRG neurons, both here and in our previous study.^41^ Furthermore, it has been found that primary afferent-evoked synaptic currents in laminae I and II, mainly originating from small peptidergic and nonpeptidergic DRG neurons, are 74% dependent on N-type channels,^3^ highlighting the preferential synaptic localization of these channels in vivo.

### 4.1. Effect of partial sciatic nerve ligation on distribution of Ca_V_2.2_HA in dorsal root ganglion neuron subtypes

Despite the importance of N-type calcium channels in neuropathic pain transmission,^11,52^ there are few studies examining the effect of different types of neuropathic injury on Ca_V_2.2 calcium channel levels or distribution in DRG cell bodies or primary afferent terminals. In this study in mice, we found that PSNL induced an increase of DRG Ca_V_2.2_HA expression, particularly on the cell surface, in medium and large DRG cell bodies, but not in small DRGs, in which expression is already high. Turning to the spinal cord, an anticipated finding was that there is a patchy loss of staining for Ca_V_2.2_HA in superficial laminae ipsilateral to the PSNL injury. This was paralleled by patchy loss of CGRP and IB4, which has been extensively documented previously by others,^2,55^ and attributed to resorption of neurotrophin-dependent terminals.^38^

### 4.2. Increase of Ca_V_2.2_HA in deep medial dorsal horn following partial sciatic nerve ligation and colocalization with GFRα1

An important and unexpected finding of this study is the clear increase in Ca_V_2.2_HA in the deep medial and central layers IV-V of the dorsal horn, ipsilateral to nerve injury. Here, the elevation of Ca_V_2.2_HA showed a very similar, although less extensive, pattern to the increase of the GDNF receptor GFRα1, following sensory nerve injury (Fig. 6). There was an increase in the association of Ca_V_2.2_HA and GFRα1 clusters in the ipsilateral deep dorsal horn (Fig. 8), and an increased colocalization of up-regulated Ca_V_2.2_HA and GFRα1 in large DRG neurons (Fig. 7). Our findings with respect to GFRα1 are similar to those found in a previous study, in which sciatic nerve injury caused a widespread increase in nerve fiber labelling for GFRα1 immunoreactivity in the dorsal horn, particularly in the deeper medial third to half of this region.^27^

Glial cell-line-derived neurotrophic factor family ligands (GDNF, neurturin, artemin, and persephin) interact with the GFRα receptor family (1-4, respectively) together with their coreceptor, the tyrosine kinase, Ret (REarranged during Transfection). Ret is a transmembrane protein, whereas the GFRα receptors are glycosyl phosphatidyl–inositol (GPI) anchored, as is α_2_δ-1.^13^ From single-cell RNA-sequencing studies in adult mouse DRG neurons,^53^ 3 clusters of large-diameter LTMRs were identified; of which, 2 were Ret positive. Ret-positive A-LTMRs have large neuronal somata, and their central endings are in deep layers of the dorsal horn.^10,32^ The A-LTMR DRGs give rise to the major tactile receptors in skin,^32^ and under pathological conditions such as neuropathic injury, A-LTMRs can also mediate the sensation of pain induced by touch, termed mechanical allodynia.^16,33^ The increase in Ret following sensory nerve injury has been found previously to be mainly in primary afferents.^26^

Together, these results indicate that the increase in Ca_V_2.2_HA expression and cell surface trafficking in cell bodies of medium and large DRG cell bodies ipsilateral to PSNL is paralleled by a similar increase in the presumed terminals of these DRGs in the deep dorsal horn. These medium and large DRG neurons and their central projections are likely to represent Aβ LTMRs that contain GFRα1,^5^ which is also up-regulated in these neurons following sensory nerve injury.^27^

### 4.3. Importance of α_2_δ-1 in redistribution of Ca_V_2.2_HA

The α_2_δ-1 auxiliary subunit associated with Ca_V_1 and Ca_V_2 calcium channels has been shown to be important for calcium channel trafficking in expression systems^9^ and in vivo*.*^41^ In both rats and mice, α_2_δ-1 protein is expressed in all DRG neurons, with highest expression in the somata of small DRG neurons.^4,41^ α_2_δ-1 plays a major role in primary afferent pain pathways and is up-regulated in all injured DRGs following neuropathic injury.^4,17,31,35,40^ Furthermore, genetic ablation of the *Cacna2d1* gene, encoding α_2_δ-1, caused a marked delay in the development of neuropathic mechanical hypersensitivity,^44^ and overexpression of α_2_δ-1 mimics features of neuropathic injury.^31^ Our previous results using mice in which α_2_δ-1 is globally ablated, crossed with Ca_V_2.2_HA^KI/KI^ mice, have emphasised an essential role of α_2_δ-1 in trafficking Ca_V_2.2_HA, both to the plasma membrane of DRG neuron cell bodies and to their primary afferent terminals in the dorsal horn.^41^ For this reason, we compared the effect of PSNL on Ca_V_2.2_HA distribution, in both α_2_δ-1^+/+^ and α_2_δ-1^−/−^ mice. We found that α_2_δ-1 knockout prevented the PSNL-induced increase in Ca_V_2.2_HA in medium and large DRG cell bodies, ipsilateral to the ligation (Figs. 1B and C), and it also prevented or markedly reduced the PSNL-induced increase in Ca_V_2.2_HA in the medial and central deep dorsal horn (Fig. 4D). By contrast, the reduction of Ca_V_2.2_HA, CGRP, and IB4 in the superficial laminae, ipsilateral to PSNL, was not affected by α_2_δ-1 knockout. Therefore, the resorption of neurotrophin-dependent terminals that underlies this patchy loss of glomerular synapses^2,38,55^ is a neuroanatomical consequence of the nerve injury that is independent of α_2_δ-1.

Furthermore, the increase in GFRα1 in DRGs and in deep dorsal horn ipsilateral to PSNL was not reduced by α_2_δ-1 knockout. This differential lack of effect of α_2_δ-1 knockout on the increase iGFRα1, relative to its effect on the Ca_V_2.2_HA increase ipsilateral to PSNL in large DRG neurons and in the deep dorsal horn, strongly suggests that the increase in Ca_V_2.2_HA in the deep dorsal horn is due to an increase in α_2_δ-1–mediated trafficking of the channel complex to the cell surface and into terminal zones, with a consequent reduction in its intracellular degradation (for review see Ref. 18). In agreement with this, no increase in *Cacna1b* mRNA has been reported following nerve injury in multiple studies, which have reported a consistent increase in expression of *Cacna2d1* mRNA.^14,35,54,58,59^

In contrast to these results using a highly specific anti-HA antibody that shows no signal in WT tissue, all previous studies examining the distribution of Ca_V_2.2 in both DRGs and dorsal horn, following several different nerve injury models, have used antipeptide antibodies against intracellular epitopes. These studies have produced varying results, using both immunohistochemistry and western blotting.^12,29,60,61^ In 2 studies, an elevation of Ca_V_2.2 immunoreactivity was observed in the dorsal horn superficial layers,^12,61^ and none revealed any elevation of Ca_V_2.2 in deeper dorsal horn. In general, the use of antipeptide antibodies, which are often of relatively low affinity and have not been previously validated in knockout mouse tissue, may produce false-positive or false-negative results, especially when directed against low-abundance proteins such as ion channels.^25^

In conclusion, the use of Ca_V_2.2_HA knockin mice has provided novel insights into alterations in Ca_V_2.2 distribution and trafficking following PSNL. Notably, we find that Ca_V_2.2_HA is up-regulated, ipsilateral to PSNL, particularly in large GFRα1-positive DRG neurons, and in parallel, there is an increase of Ca_V_2.2_HA in the ipsilateral medial and central deep dorsal horn, where GFRα1 is also up-regulated. The up-regulation of Ca_V_2.2_HA is α_2_δ-1 dependent, whereas the increase in GFRα1 is not, indicating that there is an increase in Ca_V_2.2_HA trafficking into these mechanoreceptor terminals, which is likely to mediate increased neurotransmission.

## Conflict of interest statement

The authors have no conflict of interest to declare.

## Appendix A. Supplemental digital content

Supplemental digital content associated with this article can be found online at http://links.lww.com/PAIN/B762.

## Supplemental video content

A video abstract associated with this article can be found at http://links.lww.com/PAIN/B763.

## References

[1] Albensi BC Ryujin KT McIntosh JM Naisbitt SR Olivera BM Fillous F. Localization of [^125^I]omega-conotoxin GVIA binding in human hippocampus and cerebellum. Neuroreport 1993;4:1331–4. 8260616 10.1097/00001756-199309150-00011

[2] Bailey AL Ribeiro-da-Silva A. Transient loss of terminals from non-peptidergic nociceptive fibers in the substantia gelatinosa of spinal cord following chronic constriction injury of the sciatic nerve. Neuroscience 2006;138:675–90. 16413131 10.1016/j.neuroscience.2005.11.051

[3] Bao J Li JJ Perl ER. Differences in Ca2+ channels governing generation of miniature and evoked excitatory synaptic currents in spinal laminae I and II. J Neurosci 1998;18:8740–50. 9786981 10.1523/JNEUROSCI.18-21-08740.1998 PMC6793560

[4] Bauer CS Nieto-Rostro M Rahman W Tran-Van-Minh A Ferron L Douglas L Kadurin I Sri Ranjan Y Fernandez-Alacid L Millar NS Dickenson AH Lujan R Dolphin AC. The increased trafficking of the calcium channel subunit α2δ-1 to presynaptic terminals in neuropathic pain is inhibited by the α2δ ligand pregabalin. J Neurosci 2009;29:4076–88. 19339603 10.1523/JNEUROSCI.0356-09.2009 PMC6665374

[5] Bennett DLH Boucher TJ Armanini MP Poulsen KT Michael GJ Priestley JV Phillips HS McMahon SB Shelton DL. The glial cell line-derived neurotrophic factor family receptor components are differentially regulated within sensory neurons after nerve injury. J Neurosci 2000;20:427–37. 10627618 10.1523/JNEUROSCI.20-01-00427.2000 PMC6774134

[6] Boland LM Morrill JA Bean BP. omega-Conotoxin block of N-type calcium channels in frog and rat sympathetic neurons. J Neurosci 1994;14:5011–27. 8046465 10.1523/JNEUROSCI.14-08-05011.1994 PMC6577193

[7] Boucher TJ Okuse K Bennett DLH Munson JB Wood JN McMahon SB. Potent analgesic effects of GDNF in neuropathic pain states. Science 2000;290:124–7. 11021795 10.1126/science.290.5489.124

[8] Bowersox SS Gadbois T Singh T Pettus M Wang YX Luther RR. Selective N-type neuronal voltage-sensitive calcium channel blocker, SNX-111, produces spinal antinociception in rat models of acute, persistent and neuropathic pain. J Pharmacol Exp Ther 1996;279:1243–9. 8968347

[9] Cassidy JS Ferron L Kadurin I Pratt WS Dolphin AC. Functional exofacially tagged N-type calcium channels elucidate the interaction with auxiliary α _2_ δ-1 subunits. Proc Natl Acad Sci U S A 2014;111:8979–84. 24889613 10.1073/pnas.1403731111 PMC4066493

[10] Chamessian A Matsuda M Young M Wang M Zhang ZJ Liu D Tobin B Xu ZZ Van de Ven T Ji RR. Is optogenetic activation of vglut1-positive abeta low-threshold mechanoreceptors sufficient to induce tactile allodynia in mice after nerve injury? J Neurosci 2019;39:6202–15. 31152125 10.1523/JNEUROSCI.2064-18.2019 PMC6668198

[11] Chaplan SR Pogrel JW Yaksh TL. Role of voltage-dependent calcium channel subtypes in experimental tactile allodynia. J Pharmacol Exp Ther 1994;269:1117–23. 8014856

[12] Cizkova D Marsala J Lukacova N Marsala M Jergova S Orendacova J Yaksh TL. Localization of N-type Ca2+ channels in the rat spinal cord following chronic constrictive nerve injury. Exp Brain Res 2002;147:456–63. 12444477 10.1007/s00221-002-1217-3

[13] Davies A Kadurin I Alvarez-Laviada A Douglas L Nieto-Rostro M Bauer CS Pratt WS Dolphin AC. The α2δ subunits of voltage-gated calcium channels form GPI-anchored proteins, a post-translational modification essential for function. Proc Natl Acad Sci U S A 2010;107:1654–9. 20080692 10.1073/pnas.0908735107 PMC2824380

[14] Davis-Taber RA Scott VE. Transcriptional profiling of dorsal root gangliain a neuropathic pain model using microarrayand laser capture microdissection. Drug Develop Res 2006;67:308–30.

[15] Desmadryl G Hilaire C Vigues S Diochot S Valmier J. Developmental regulation of T-N- and L-type calcium currents in mouse embryonic sensory neurones. Eur J Neurosci 1998;10:545–52. 9749717 10.1046/j.1460-9568.1998.00055.x

[16] Devor M. Ectopic discharge in Aβ afferents as a source of neuropathic pain. Exp Brain Res 2009;196:115–28. 19242687 10.1007/s00221-009-1724-6

[17] Dolphin AC. Calcium channel auxiliary α2δ and β subunits: trafficking and one step beyond. Nat Rev Neurosci 2012;13:542–55. 22805911 10.1038/nrn3311

[18] Dolphin AC Lee A. Presynaptic calcium channels: specialized control of synaptic neurotransmitter release. Nat Rev Neurosci 2020;21:213–29. 32161339 10.1038/s41583-020-0278-2 PMC7873717

[19] Emery EC Luiz AP Sikandar S Magnusdottir R Dong X Wood JN. In vivo characterization of distinct modality-specific subsets of somatosensory neurons using GCaMP. Sci Adv 2016;2:e1600990. 27847865 10.1126/sciadv.1600990 PMC5106201

[20] Fox AP Nowycky MC Tsien RW. Single-channel recordings of three types of calcium channels in chick sensory neurones. J Physiol 1987;394:173–200. 2451017 10.1113/jphysiol.1987.sp016865 PMC1191956

[21] Fuller-Bicer GA Varadi G Koch SE Ishii M Bodi I Kadeer N Muth JN Mikala G Petrashevskaya NN Jordan MA Zhang SP Qin N Flores CM Isaacsohn I Varadi M Mori Y Jones WK Schwartz A. Targeted disruption of the voltage-dependent calcium channel alpha2/delta-1-subunit. Am J Physiol Heart Circ Physiol 2009;297:H117–24. 19429829 10.1152/ajpheart.00122.2009 PMC2711723

[22] Gohil K Bell JR Ramachandran J Miljanich GP. Neuroanatomical distribution of receptors for a novel voltage-sensitive calcium-channel antagonist, SNX-230 (omega-conopeptide MVIIC). Brain Res 1994;653:258–66. 7982059 10.1016/0006-8993(94)90398-0

[23] Grantham CJ Bowman D Bath CP Bell DC Bleakman D. ω-conotoxin MVIIC reversibly inhibits a human N-type calcium channel and calcium influx into chick synaptosomes. Neuropharmacology 1994;33:255–8. 8035912 10.1016/0028-3908(94)90017-5

[24] Hirning LD Fox AP McCleskey EW Olivera BM Thayer SA Miller RJ Tsien RW. Dominant role of N-type Ca^2+^ channels in evoked release of norepinephrine from sympathetic neurons. Science 1988;239:57–61. 2447647 10.1126/science.2447647

[25] Ivell R Teerds K Hoffman GE. Proper application of antibodies for immunohistochemical detection: antibody crimes and how to prevent them. Endocrinology 2014;155:676–87. 24428532 10.1210/en.2013-1971 PMC3929726

[26] Jongen JL Jaarsma D Hossaini M Natarajan D Haasdijk ED Holstege JC. Distribution of RET immunoreactivity in the rodent spinal cord and changes after nerve injury. J Comp Neurol 2007;500:1136–53. 17183535 10.1002/cne.21234

[27] Keast JR Forrest SL Osborne PB. Sciatic nerve injury in adult rats causes distinct changes in the central projections of sensory neurons expressing different glial cell line-derived neurotrophic factor family receptors. J Comp Neurol 2010;518:3024–45. 20533358 10.1002/cne.22378 PMC2883785

[28] Kim C Jun K Lee T Kim SS McEnery MW Chin H Kim HL Park JM Kim DK Jung SJ Kim J Shin HS. Altered nociceptive response in mice deficient in the α_1B_ subunit of the voltage-dependent calcium channel. Mol Cell Neurosci 2001;18:235–45. 11520183 10.1006/mcne.2001.1013

[29] Leo M Schmitt LI Jastrow H Thomale J Kleinschnitz C Hagenacker T. Cisplatin alters the function and expression of N-type voltage-gated calcium channels in the absence of morphological damage of sensory neurons. Mol Pain 2017;13:174480691774656. 10.1177/1744806917746565 PMC5731623 29166837

[30] Li CY Song YH Higuera ES Luo ZD. Spinal dorsal horn calcium channel alpha2delta-1 subunit upregulation contributes to peripheral nerve injury-induced tactile allodynia. J Neurosci 2004;24:8494–9. 15456823 10.1523/JNEUROSCI.2982-04.2004 PMC1635787

[31] Li CY Zhang XL Matthews EA Li KW Kurwa A Boroujerdi A Gross J Gold MS Dickenson AH Feng G Luo DZ. Calcium channel α2δ1 subunit mediates spinal hyperexcitability in pain modulation. PAIN 2006;125:20–34. 16764990 10.1016/j.pain.2006.04.022 PMC1635965

[32] Li L Rutlin M Abraira VE Cassidy C Kus L Gong S Jankowski MP Luo W Heintz N Koerber HR Woodbury CJ Ginty DD. The functional organization of cutaneous low-threshold mechanosensory neurons. Cell 2011;147:1615–27. 22196735 10.1016/j.cell.2011.11.027 PMC3262167

[33] Lolignier S Eijkelkamp N Wood JN. Mechanical allodynia. Pflügers Archiv 2015;467:133–9. 24846747 10.1007/s00424-014-1532-0 PMC4281368

[34] Luo ZD Calcutt NA Higuera ES Valder CR Song YH Svensson CI Myers RR. Injury type-specific calcium channel alpha 2 delta-1 subunit up-regulation in rat neuropathic pain models correlates with antiallodynic effects of gabapentin. J Pharmacol Exp Ther 2002;303:1199–205. 12438544 10.1124/jpet.102.041574

[35] Luo ZD Chaplan SR Higuera ES Sorkin LS Stauderman KA Williams ME Yaksh TL. Upregulation of dorsal root ganglion α_2_δ calcium channel subunit and its correlation with allodynia in spinal nerve-injured rats. J Neurosci 2001;21:1868–75. 11245671 10.1523/JNEUROSCI.21-06-01868.2001 PMC6762626

[36] McGivern JG McDonough SI. Voltage-gated calcium channels as targets for the treatment of chronic pain. Curr Drug Targets CNS Neurol Disord 2004;3:457–78. 15578964 10.2174/1568007043336743

[37] Miljanich GP. Ziconotide: neuronal calcium channel blocker for treating severe chronic pain. Curr Med Chem 2004;11:3029–40. 15578997 10.2174/0929867043363884

[38] Molander C Wang HF Rivero-Melian C Grant G. Early decline and late restoration of spinal cord binding and transganglionic transport of isolectin B4 from Griffonia simplicifolia I after peripheral nerve transection or crush. Restorative Neurol Neurosci 1996;10:123–33. 10.3233/RNN-1996-10301 21551513

[39] Murali SS Napier IA Mohammadi SA Alewood PF Lewis RJ Christie MJ. High-voltage-activated calcium current subtypes in mouse DRG neurons adapt in a subpopulation-specific manner after nerve injury. J Neurophysiol 2015;113:1511–9. 25505111 10.1152/jn.00608.2014

[40] Newton RA Bingham S Case PC Sanger GJ Lawson SN. Dorsal root ganglion neurons show increased expression of the calcium channel α2δ-1 subunit following partial sciatic nerve injury. Mol Brain Res 2001;95:1–8. 11687271 10.1016/s0169-328x(01)00188-7

[41] Nieto-Rostro M Ramgoolam K Pratt WS Kulik A Dolphin AC. Ablation of α_2_δ-1 inhibits cell-surface trafficking of endogenous N-type calcium channels in the pain pathway in vivo. Proc Natl Acad Sci U S A 2018;115:E12043–52. 30487217 10.1073/pnas.1811212115 PMC6305000

[42] Nowycky MC Fox AP Tsien RW. Three types of neuronal calcium channel with different calcium agonist sensitivity. Nature 1985;316:440–3. 2410796 10.1038/316440a0

[43] Pajouhesh H Feng ZP Zhang L Pajouhesh H Jiang X Hendricson A Dong H Tringham E Ding Y Vanderah TW Porreca F Belardetti F Zamponi GW Mitscher LA Snutch TP. Structure-activity relationships of trimethoxybenzyl piperazine N-type calcium channel inhibitors. Bioorg Med Chem Lett 2012;22:4153–8. 22579422 10.1016/j.bmcl.2012.04.054

[44] Patel R Bauer CS Nieto-Rostro M Margas W Ferron L Chaggar K Crews K Ramirez JD Bennett DLH Schwartz A Dickenson AH Dolphin AC. α2δ-1 gene deletion affects somatosensory neuron function and delays mechanical hypersensitivity in response to peripheral nerve damage. J Neurosci 2013;33:16412–26. 24133248 10.1523/JNEUROSCI.1026-13.2013 PMC3797367

[45] Patel R Montagut-Bordas C Dickenson AH. Calcium channel modulation as a target in chronic pain control. Br J Pharmacol 2018;175:2173–84. 28320042 10.1111/bph.13789 PMC5980588

[46] Plummer MR Logothetis DE Hess P. Elementary properties and pharmacological sensitivities of calcium channels in mammalian peripheral neurons. Neuron 1989;2:1453–63. 2560643 10.1016/0896-6273(89)90191-8

[47] Saegusa H Kurihara T Zong S Kazuno A Matsuda Y Nonaka T Han W Toriyama H Tanabe T. Suppression of inflammatory and neuropathic pain symptoms in mice lacking the N-type Ca^2+^ channel. EMBO J 2001;20:2349–56. 11350923 10.1093/emboj/20.10.2349 PMC125247

[48] Scroggs RS Fox AP. Distribution of dihydropyridine and omega-conotoxin-sensitive calcium currents in acutely isolated rat and frog sensory neuron somata: diameter-dependent L channel expression in frog. J Neurosci 1991;11:1334–46. 1709205 10.1523/JNEUROSCI.11-05-01334.1991 PMC6575325

[49] Scroggs RS Fox AP. Calcium current variation between acutely isolated adult rat dorsal root ganglion neurons of different size. J Physiol 1992;445:639–58. 1323671 10.1113/jphysiol.1992.sp018944 PMC1180002

[50] Seltzer Z Dubner R Shir Y. A novel behavioral model of neuropathic pain disorders produced in rats by partial sciatic nerve injury. PAIN 1990;43:205–18. 1982347 10.1016/0304-3959(90)91074-S

[51] Staats PS Yearwood T Charapata SG Presley RW Wallace MS Byas-Smith M Fisher R Bryce DA Mangieri EA Luther RR Mayo M McGuire D Ellis D. Intrathecal ziconotide in the treatment of refractory pain in patients with cancer or AIDS: a randomized controlled trial. JAMA 2004;291:63–70. 14709577 10.1001/jama.291.1.63

[52] Swensen AM Herrington J Bugianesi RM Dai G Haedo RJ Ratliff KS Smith MM Warren VA Arneric SP Eduljee C Parker D Snutch TP Hoyt SB London C Duffy JL Kaczorowski GJ McManus OB. Characterization of the substituted N-triazole oxindole TROX-1, a small-molecule, state-dependent inhibitor of Ca(V)2 calcium channels. Mol Pharmacol 2012;81:488–97. 22188924 10.1124/mol.111.075226

[53] Usoskin D Furlan A Islam S Abdo H Lonnerberg P Lou D Hjerling-Leffler J Haeggstrom J Kharchenko O Kharchenko PV Linnarsson S Ernfors P. Unbiased classification of sensory neuron types by large-scale single-cell RNA sequencing. Nat Neurosci 2015;18:145–53. 25420068 10.1038/nn.3881

[54] Wang H Sun H Della Penna K Benz RJ Xu J Gerhold DL Holder DJ Koblan KS. Chronic neuropathic pain is accompanied by global changes in gene expression and shares pathobiology with neurodegenerative diseases. Neuroscience 2002;114:529–46. 12220557 10.1016/s0306-4522(02)00341-x

[55] Wang R Guo W Ossipov MH Vanderah TW Porreca F Lai J. Glial cell line-derived neurotrophic factor normalizes neurochemical changes in injured dorsal root ganglion neurons and prevents the expression of experimental neuropathic pain. Neuroscience 2003;121:815–24. 14568039 10.1016/s0306-4522(03)00491-3

[56] Wang T Molliver DC Jing X Schwartz ES Yang FC Samad OA Ma Q Davis BM. Phenotypic switching of nonpeptidergic cutaneous sensory neurons following peripheral nerve injury. PLoS One 2011;6:e28908. 22216140 10.1371/journal.pone.0028908 PMC3244441

[57] Wang YX Pettus M Gao D Phillips C Scott Bowersox S. Effects of intrathecal administration of ziconotide, a selective neuronal N-type calcium channel blocker, on mechanical allodynia and heat hyperalgesia in a rat model of postoperative pain. PAIN 2000;84:151–8. 10666519 10.1016/s0304-3959(99)00197-9

[58] Wu S Marie Lutz B Miao X Liang L Mo K Chang YJ Du P Soteropoulos P Tian B Kaufman AG Bekker A Hu Y Tao YX. Dorsal root ganglion transcriptome analysis following peripheral nerve injury in mice. Mol Pain 2016;12:174480691662904. 10.1177/1744806916629048 PMC4955972 27030721

[59] Xiao HS Huang QH Zhang FX Bao L Lu YJ Guo C Yang L Huang WJ Fu G Xu SH Cheng XP Yan Q Zhu ZD Zhang X Chen Z Han ZG Zhang X. Identification of gene expression profile of dorsal root ganglion in the rat peripheral axotomy model of neuropathic pain. Proc Natl Acad Sci U S A 2002;99:8360–5. 12060780 10.1073/pnas.122231899 PMC123072

[60] Yang J Xie MX Hu L Wang XF Mai JZ Li YY Wu N Zhang C Li J Pang RP Liu XG. Upregulation of N-type calcium channels in the soma of uninjured dorsal root ganglion neurons contributes to neuropathic pain by increasing neuronal excitability following peripheral nerve injury. Brain Behav Immun 2018;71:52–65. 29709527 10.1016/j.bbi.2018.04.016

[61] Yu H Shin SM Xiang H Chao D Cai Y Xu H Khanna R Pan B Hogan QH. AAV-encoded CaV2.2 peptide aptamer CBD3A6K for primary sensory neuron-targeted treatment of established neuropathic pain. Gene Ther 2019;26:308–23. 31118475 10.1038/s41434-019-0082-7 PMC6707887

[62] Zimmermann M. Ethical guidelines for investigations of experimental pain in conscious animals. PAIN 1983;16:109–10. 6877845 10.1016/0304-3959(83)90201-4

