# Supplementary material for: Nerve injury increases native CaV2.2 trafficking in dorsal root ganglion mechanoreceptors
Source: Pain. 2022 Dec 15;164(6):1264–79. doi: 10.1097/j.pain.0000000000002846 (PMC10184561; doi:10.1097/j.pain.0000000000002846)
Supplement: Supplementary file 1 [file jop-164-1264-s001.pdf]

## Supplementary Table 1

Two-way ANOVA for data in Figs. 4C and D, separated according to sex

| Table Analyzed | Superficial Medial | | | | |
|---|---|---|---|---|---|
| | | | | | |
| Two-way ANOVA | Ordinary | | | | |
| Alpha | 0.05 | | | | |
| | | | | | |
| Source of Variation | % of total | P value | P value summary | Significant? | |
| Interaction | 4.282 | 0.4118 | ns | No | |
| genotype | 1.44 | 0.6314 | ns | No | |
| sex | 1.871 | 0.585 | ns | No | |
| | | | | | |
| ANOVA table | SS (Type | DF | MS | F (DFn, DFd) | P value |
| Interaction | 0.00855 | 1 | 0.008545 | F (1, 15) = 0.7128 | P=0.4118 |
| genotype | 0.00288 | 1 | 0.002875 | F (1, 15) = 0.2398 | P=0.6314 |
| sex | 0.00373 | 1 | 0.003734 | F (1, 15) = 0.3114 | P=0.5850 |
| Residual | 0.1798 | 15 | 0.01199 | | |
| | Superficial Central | | | | |
| | | | | | |
| Two-way ANOVA | Ordinary | | | | |
| Alpha | 0.05 | | | | |
| | | | | | |
| Source of Variation | % of total | P value | P value summary | Significant? | |
| Interaction | 2.429 | 0.517 | ns | No | |
| genotype | 9.05 | 0.2197 | ns | No | |
| sex | 3.595 | 0.432 | ns | No | |
| | | | | | |
| ANOVA table | SS (Type | DF | MS | F (DFn, DFd) | P value |
| Interaction | 0.00355 | 1 | 0.003552 | F (1, 15) = 0.4404 | P=0.5170 |
| genotype | 0.01323 | 1 | 0.01323 | F (1, 15) = 1.641 | P=0.2197 |
| sex | 0.00526 | 1 | 0.005258 | F (1, 15) = 0.6519 | P=0.4320 |
| Residual | 0.121 | 15 | 0.008065 | | |
| | Superficial Lateral | | | | |
| | | | | | |
| Two-way ANOVA | Ordinary | | | | |
| Alpha | 0.05 | | | | |
| | | | | | |
| Source of Variation | % of total | P value | P value summary | Significant? | |
| Interaction | 0.2941 | 0.8354 | ns | No | |
| genotype | 0.00842 | 0.9719 | ns | No | |
| sex | 1.251 | 0.6691 | ns | No | |
| | | | | | |
| ANOVA table | SS (Type | DF | MS | F (DFn, DFd) | P value |
| Interaction | 0.00052 | 1 | 0.0005167 | F (1, 15) = 0.04471 | P=0.8354 |
| genotype | 1.5E-05 | 1 | 0.00001479 | F (1, 15) = 0.00128 | P=0.9719 |
| sex | 0.0022 | 1 | 0.002197 | F (1, 15) = 0.1901 | P=0.6691 |
| Residual | 0.1733 | 15 | 0.01156 | | |

| Table Analyzed | Deep Medial | | | | |
|---|---|---|---|---|---|
| | | | | | |
| Two-way ANOVA | Ordinary | | | | |
| Alpha | 0.05 | | | | |
| | | | | | |
| Source of Variation | % of total | P value | P value summary | Significant? | |
| Interaction | 0.543 | 0.7464 | ns | No | |
| genotype | 23.46 | 0.0469 | * | Yes | |
| sex | 0.1343 | 0.8721 | ns | No | |
| | | | | | |
| ANOVA table | SS (Type | DF | MS | F (DFn, DFd) | P value |
| Interaction | 0.001 | 1 | 0.001002 | F (1, 15) = 0.1085 | P=0.7464 |
| genotype | 0.04331 | 1 | 0.04331 | F (1, 15) = 4.688 | P=0.0469 |
| sex | 0.00025 | 1 | 0.0002478 | F (1, 15) = 0.02683 | P=0.8721 |
| Residual | 0.1386 | 15 | 0.009238 | | |
| | Deep Central | | | | |
| | | | | | |
| Two-way ANOVA | Ordinary | | | | |
| Alpha | 0.05 | | | | |
| | | | | | |
| Source of Variation | % of total | P value | P value summary | Significant? | |
| Interaction | 0.00137 | 0.9855 | ns | No | |
| genotype | 30.95 | 0.0143 | * | Yes | |
| sex | 5.797 | 0.2489 | ns | No | |
| | | | | | |
| ANOVA table | SS (Type | DF | MS | F (DFn, DFd) | P value |
| Interaction | 2.2E-06 | 1 | 0.000002201 | F (1, 15) = 0.000339 | P=0.9855 |
| genotype | 0.04984 | 1 | 0.04984 | F (1, 15) = 7.682 | P=0.0143 |
| sex | 0.00934 | 1 | 0.009335 | F (1, 15) = 1.439 | P=0.2489 |
| Residual | 0.09732 | 15 | 0.006488 | | |
| | Deep Lateral | | | | |
| | | | | | |
| Two-way ANOVA | Ordinary | | | | |
| Alpha | 0.05 | | | | |
| | | | | | |
| Source of Variation | % of total | P value | P value summary | Significant? | |
| Interaction | 8.449 | 0.1877 | ns | No | |
| genotype | 17.89 | 0.0629 | ns | No | |
| sex | 3.422 | 0.3935 | ns | No | |
| | | | | | |
| ANOVA table | SS (Type | DF | MS | F (DFn, DFd) | P value |
| Interaction | 0.00566 | 1 | 0.005656 | F (1, 15) = 1.905 | P=0.1877 |
| genotype | 0.01198 | 1 | 0.01198 | F (1, 15) = 4.035 | P=0.0629 |
| sex | 0.00229 | 1 | 0.002291 | F (1, 15) = 0.7717 | P=0.3935 |
| Residual | 0.04453 | 15 | 0.002969 | | |

## Details of statistical test results

| Fig 3.D | | ANOVA table | SS | DF | MS | F (DFn, DFd) | P value |
|---|---|---|---|---|---|---|---|
| PSNL CaV2.2_HAKI/KI α2δ-1+/+ | HA | Size (Bin) | 25327 | 14 | 1809 | F (14, 56) = 248.3 | P<0.0001 |
| 5 sections, HA & IB4 & CGRP | | Side (ipsi or contra) | 1485 | 1 | 1485 | F (1, 4) = 14.01 | P=0.0201 |
| Superficial Dorsal Horn | | Size x Side | 1196 | 14 | 85.43 | F (14, 56) = 5.335 | P<0.0001 |
| | | | | | | | |
| | | Intensity (Bin) | 37170 | 10 | 3717 | F (10, 40) = 103.3 | P<0.0001 |
| | | Side (ipsi or contra) | 2402 | 1 | 2402 | F (1, 4) = 13.33 | P=0.0217 |
| | | Intensity x Side | 5670 | 10 | 567 | F (10, 40) = 12.66 | P<0.0001 |
| | | | | | | | |
| | IB4 | Size (Bin) | 4390 | 14 | 313.6 | F (14, 56) = 55.98 | P<0.0001 |
| | | Side (ipsi or contra) | 902.8 | 1 | 902.8 | F (1, 4) = 27.23 | P=0.0064 |
| | | Size x Side | 1244 | 14 | 88.84 | F (14, 56) = 8.002 | P<0.0001 |
| | | | | | | | |
| | | Intensity (Bin) | 4005 | 10 | 400.5 | F (10, 40) = 20.15 | P<0.0001 |
| | | Side (ipsi or contra) | 1039 | 1 | 1039 | F (1, 4) = 27.16 | P=0.0065 |
| | | Intensity x Side | 1651 | 10 | 165.1 | F (10, 40) = 14.65 | P<0.0001 |
| | | | | | | | |
| | CGRP | Size (Bin) | 5336 | 14 | 381.2 | F (14, 56) = 85.55 | P<0.0001 |
| | | Side (ipsi or contra) | 592 | 1 | 592 | F (1, 4) = 30.22 | P=0.0053 |
| | | Size x Side | 808 | 14 | 57.71 | F (14, 56) = 10.75 | P<0.0001 |
| | | | | | | | |
| | | Intensity (Bin) | 3149 | 10 | 314.9 | F (10, 40) = 38.31 | P<0.0001 |
| | | Side (ipsi or contra) | 823.6 | 1 | 823.6 | F (1, 4) = 29.79 | P=0.0055 |
| | | Intensity x Side | 799.1 | 10 | 79.91 | F (10, 40) = 12.24 | P<0.0001 |

| Fig 8.D | | ANOVA table | SS | DF | MS | F (DFn, DFd) | P value |
|---|---|---|---|---|---|---|---|
| PSNL CaV2.2_HAKI/KI α2δ-1+/+ | HA | Size (Bin) | 33842 | 14 | 2417 | F (14, 70) = 175.3 | P<0.0001 |
| 6 sections, HA & GFRα1 | | Side (ipsi or contra) | 2509 | 1 | 2509 | F (1, 5) = 14.95 | P=0.0118 |
| Superficial Dorsal Horn | | Size x Side | 2783 | 14 | 198.8 | F (14, 70) = 8.559 | P<0.0001 |
| | | | | | | | |
| | | Intensity (Bin) | 33581 | 10 | 3358 | F (10, 50) = 47.48 | P<0.0001 |
| | | Side (ipsi or contra) | 3680 | 1 | 3680 | F (1, 5) = 15.64 | P=0.0108 |
| | | Intensity x Side | 6747 | 10 | 674.7 | F (10, 50) = 9.984 | P<0.0001 |
| | | | | | | | |
| | GFRa1 | Size (Bin) | 43679 | 14 | 3120 | F (14, 70) = 193.7 | P<0.0001 |
| | | Side (ipsi or contra) | 1095 | 1 | 1095 | F (1, 5) = 72.69 | P=0.0004 |
| | | Size x Side | 3062 | 14 | 218.7 | F (14, 70) = 22.96 | P<0.0001 |
| | | | | | | | |
| | | Intensity (Bin) | 40477 | 10 | 4048 | F (10, 50) = 77.76 | P<0.0001 |
| | | Side (ipsi or contra) | 1487 | 1 | 1487 | F (1, 5) = 70.89 | P=0.0004 |
| | | Intensity x Side | 4067 | 10 | 406.7 | F (10, 50) = 18.08 | P<0.0001 |
| Sup. Fig 6.C | | | | | | | |
| PSNL CaV2.2_HAKI/KI α2δ-1+/+ | HA | Size (Bin) | 17109 | 14 | 1222 | F (14, 70) = 75.50 | P<0.0001 |
| 6 sections, HA & GFRα1 | | Side (ipsi or contra) | 2614 | 1 | 2614 | F (1, 5) = 32.90 | P=0.0023 |
| Deeper Dorsal horn | | Size x Side | 7529 | 14 | 537.8 | F (14, 70) = 24.41 | P<0.0001 |
| | | | | | | | |
| | | Intensity (Bin) | 26627 | 11 | 2421 | F (11, 55) = 15.89 | P<0.0001 |
| | | Side (ipsi or contra) | 3306 | 1 | 3306 | F (1, 5) = 32.78 | P=0.0023 |
| | | Intensity x Side | 15077 | 11 | 1371 | F (11, 55) = 13.60 | P<0.0001 |
| | | | | | | | |
| | GFRa1 | Size (Bin) | 30644 | 14 | 2189 | F (14, 70) = 143.6 | P<0.0001 |
| | | Side (ipsi or contra) | 8792 | 1 | 8792 | F (1, 5) = 109.1 | P=0.0001 |
| | | Size x Side | 17325 | 14 | 1237 | F (14, 70) = 43.71 | P<0.0001 |
| | | | | | | | |
| | | Intensity (Bin) | 44482 | 11 | 4044 | F (11, 55) = 11.66 | P<0.0001 |
| | | Side (ipsi or contra) | 11520 | 1 | 11520 | F (1, 5) = 111.0 | P=0.0001 |
| | | Intensity x Side | 28746 | 11 | 2613 | F (11, 55) = 10.81 | P<0.0001 |

| Sup. Fig 3.D | | ANOVA table | SS | DF | MS | F (DFn, DFd) | P value |
|---|---|---|---|---|---|---|---|
| PSNL CaV2.2_HAKI/KI α2δ-1-/- | HA | Size (Bin) | 36985 | 14 | 2642 | F (14, 70) = 58.53 | P<0.0001 |
| 5 sections, HA & IB4 & CGRP | | Side (ipsi or contra) | 1934 | 1 | 1934 | F (1, 5) = 5.259 | P=0.0704 |
| Superficial Dorsal Horn | | Size x Side | 2211 | 14 | 157.9 | F (14, 70) = 3.866 | P<0.0001 |
| | | Intensity (Bin) | 76792 | 10 | 7679 | F (10, 50) = 40.74 | P<0.0001 |
| | | Side (ipsi or contra) | 3201 | 1 | 3201 | F (1, 5) = 5.233 | P=0.0709 |
| | | Intensity x Side | 6897 | 10 | 689.7 | F (10, 50) = 5.932 | P<0.0001 |
| | IB4 | Size (Bin) | 7181 | 14 | 513 | F (14, 70) = 38.73 | P<0.0001 |
| | | Side (ipsi or contra) | 1150 | 1 | 1150 | F (1, 5) = 11.55 | P=0.0193 |
| | | Size x Side | 1258 | 14 | 89.83 | F (14, 70) = 5.960 | P<0.0001 |
| | | Intensity (Bin) | 12640 | 10 | 1264 | F (10, 50) = 42.30 | P<0.0001 |
| | | Side (ipsi or contra) | 1666 | 1 | 1666 | F (1, 5) = 11.21 | P=0.0204 |
| | | Intensity x Side | 3353 | 10 | 335.3 | F (10, 50) = 8.242 | P<0.0001 |
| | CGRP | Size (Bin) | 9564 | 14 | 683.2 | F (14, 70) = 88.29 | P<0.0001 |
| | | Side (ipsi or contra) | 680.6 | 1 | 680.6 | F (1, 5) = 5.537 | P=0.0653 |
| | | Size x Side | 1016 | 14 | 72.59 | F (14, 70) = 3.515 | P=0.0002 |
| | | Intensity (Bin) | 4246 | 10 | 424.6 | F (10, 50) = 8.189 | P<0.0001 |
| | | Side (ipsi or contra) | 970.9 | 1 | 970.9 | F (1, 5) = 5.661 | P=0.0632 |
| | | Intensity x Side | 1281 | 10 | 128.1 | F (10, 50) = 7.099 | P<0.0001 |

| Sup.Fig 7.D | | ANOVA table | SS | DF | MS | F (DFn, DFd) | P value |
|---|---|---|---|---|---|---|---|
| PSNL CaV2.2_HAKI/KI α2δ-1-/- | HA | Size (Bin) | 17084 | 14 | 1220 | F (14, 42) = 46.94 | P<0.0001 |
| 4 sections, HA & GFRα1 | | Side (ipsi or contra) | 1733 | 1 | 1733 | F (1, 3) = 4.395 | P=0.1270 |
| Superficial Dorsal Horn | | Size x Side | 2703 | 14 | 193.1 | F (14, 42) = 3.428 | P=0.0010 |
| | | Intensity (Bin) | 22019 | 10 | 2202 | F (10, 30) = 48.56 | P<0.0001 |
| | | Side (ipsi or contra) | 2596 | 1 | 2596 | F (1, 3) = 4.675 | P=0.1193 |
| | | Intensity x Side | 4498 | 10 | 449.8 | F (10, 30) = 4.771 | P=0.0004 |
| | GFRa1 | Size (Bin) | 29456 | 14 | 2104 | F (14, 42) = 35.71 | P<0.0001 |
| | | Side (ipsi or contra) | 99.01 | 1 | 99.01 | F (1, 3) = 0.9260 | P=0.4069 |
| | | Size x Side | 313.1 | 14 | 22.37 | F (14, 42) = 0.7364 | P=0.7267 |
| | | Intensity (Bin) | 20580 | 10 | 2058 | F (10, 30) = 7.574 | P<0.0001 |
| | | Side (ipsi or contra) | 140 | 1 | 140 | F (1, 3) = 0.9220 | P=0.4078 |
| | | Intensity x Side | 634.6 | 10 | 63.46 | F (10, 30) = 1.069 | P=0.4147 |
| Sup.Fig 7.E | | | | | | | |
| PSNL CaV2.2_HAKI/KI α2δ-1-/- | HA | Size (Bin) | 5125 | 14 | 366.1 | F (14, 42) = 122.1 | P<0.0001 |
| 4 sections, HA & GFRα1 | | Side (ipsi or contra) | 10.21 | 1 | 10.21 | F (1, 3) = 0.5052 | P=0.5285 |
| Deeper Dorsal horn | | Size x Side | 29.92 | 14 | 2.137 | F (14, 42) = 0.2441 | P=0.9968 |
| | | Intensity (Bin) | 5340 | 11 | 485.5 | F (11, 33) = 62.36 | P<0.0001 |
| | | Side (ipsi or contra) | 12.04 | 1 | 12.04 | F (1, 3) = 0.4669 | P=0.5435 |
| | | Intensity x Side | 25.96 | 11 | 2.36 | F (11, 33) = 0.1655 | P=0.9985 |
| | GFRa1 | Size (Bin) | 20863 | 14 | 1490 | F (14, 42) = 85.36 | P<0.0001 |
| | | Side (ipsi or contra) | 4551 | 1 | 4551 | F (1, 3) = 78.70 | P=0.0030 |
| | | Size x Side | 10152 | 14 | 725.1 | F (14, 42) = 57.64 | P<0.0001 |
| | | Intensity (Bin) | 18936 | 11 | 1721 | F (11, 33) = 2.619 | P=0.0160 |
| | | Side (ipsi or contra) | 5891 | 1 | 5891 | F (1, 3) = 75.85 | P=0.0032 |
| | | Intensity x Side | 10269 | 11 | 933.5 | F (11, 33) = 2.564 | P=0.0180 |

## Supplementary Figures

**Supplementary Fig. 1: Ca$_V$2.2_HA in individual DRG neurons from Ca$_V$2.2_HA$^{KI/KI}$ PSNL and sham-operated mice**

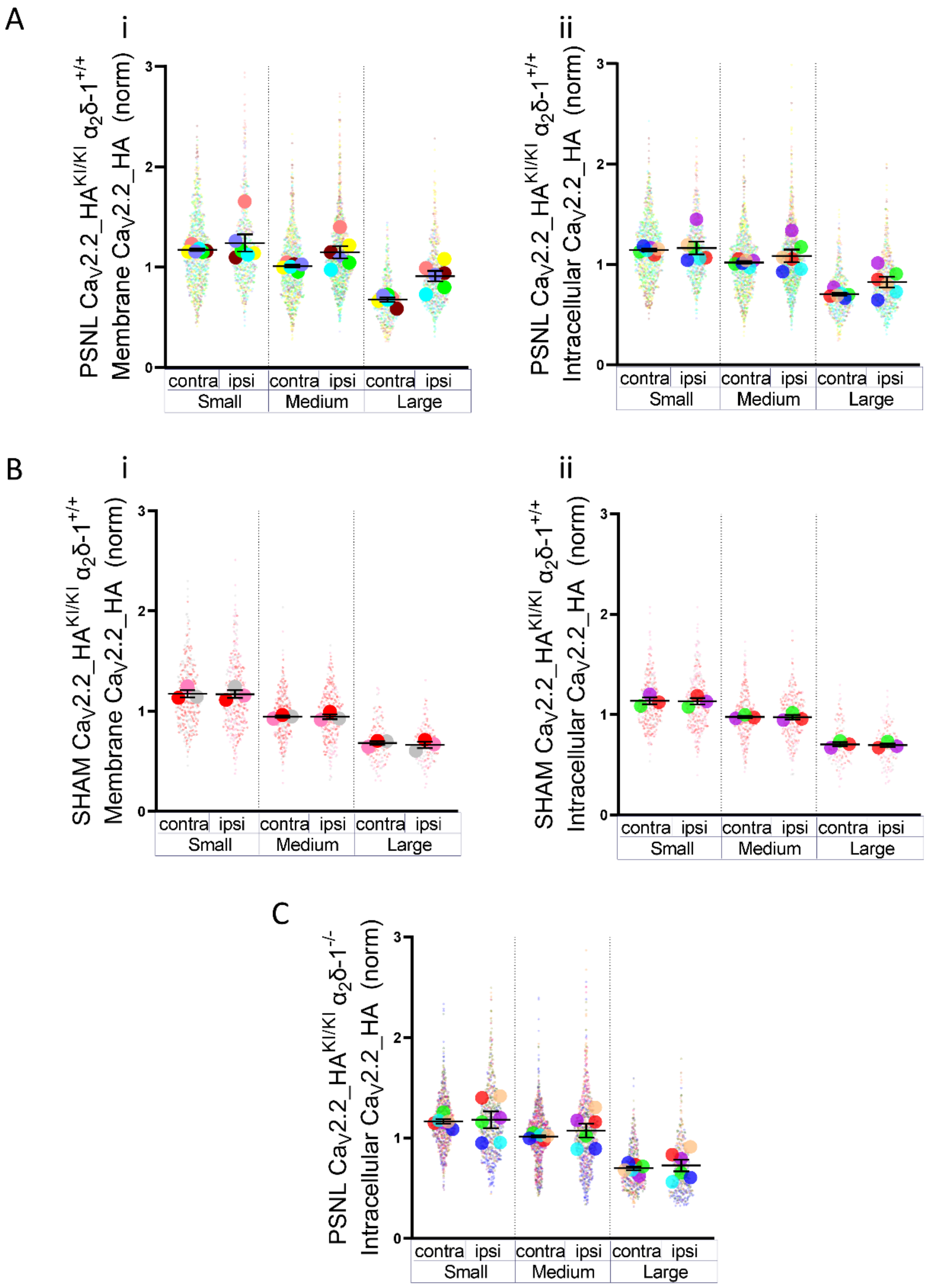


A, B: Membrane (i) and intracellular (ii) HA staining quantified with respect to cell size (small < 61 µm, medium 61-94 µm, large >94 µm perimeter) from ipsilateral (ipsi) and contralateral (contra) DRGs from (A) PSNL Ca$_V$2.2_HA$^{KI/KI}$, $\alpha_2\delta$-1 wildtype ($\alpha_2\delta$-1_WT, N= 6 mice, 2200 ipsi and 2235 contra DRGs) or (B) SHAM Ca$_V$2.2_HA$^{KI/KI}$, $\alpha_2\delta$-1 wildtype ($\alpha_2\delta$-1_WT, N=3 mice, 728 ipsi and 731 contra DRGs) . For each mouse all cells from at least 3 sections from the ipsi and contra L4 DRGs were analysed and normalised to the mean of the contra side (data from each mouse is shown in a different colour).

C: Intracellular HA staining in Ca$_V$2.2_HA$^{KI/KI}$ $\alpha_2\delta$-1$^{-/-}$ ($\alpha_2\delta$-1_KO, N=6 mice, 1980 ipsi and 2186 contra DRGs).

**Supplementary Fig. 2: Distribution of IB4 and CGRP from $Ca_V2.2\_HA^{KI/KI}$** $\alpha_2\delta$-1 WT and $\alpha_2\delta$-1 KO **mice following PSNL**

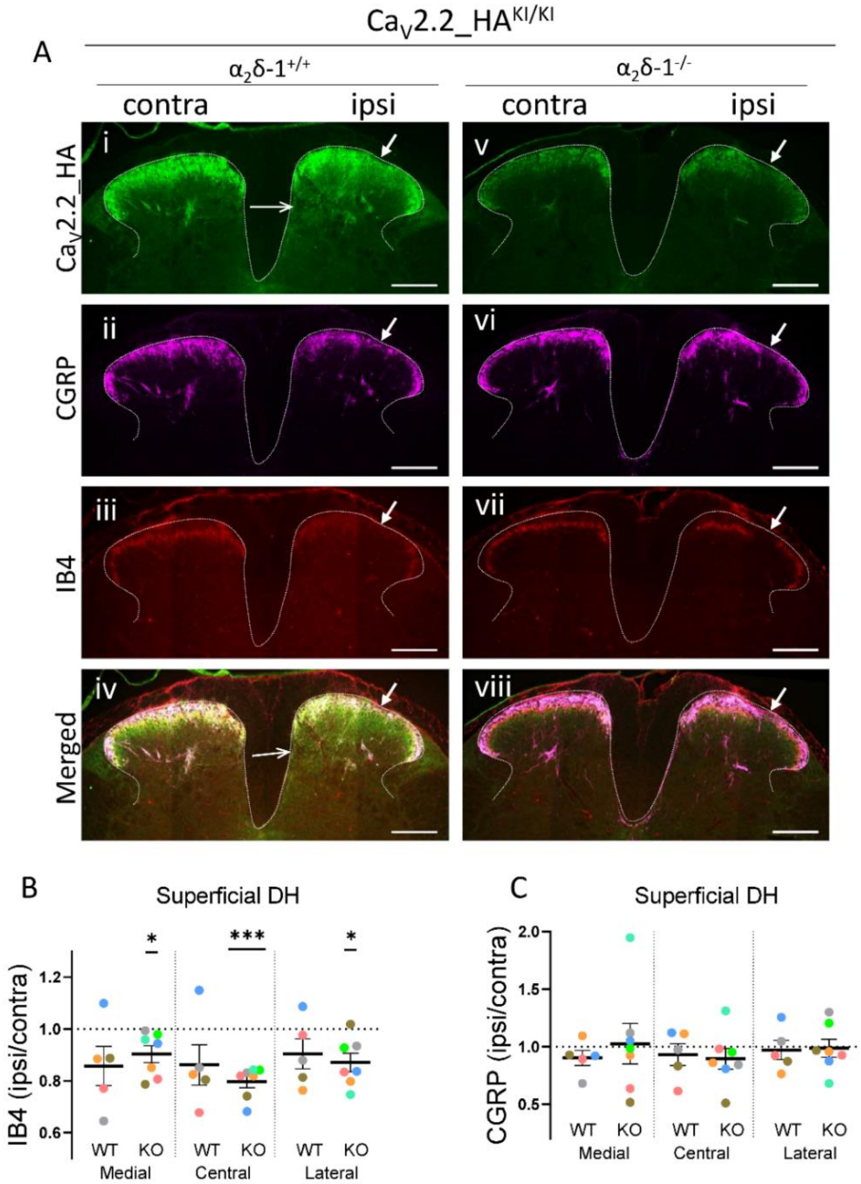


A: Representative images of dorsal horn sections following PSNL, in $Ca_V2.2\_HA^{KI/KI}$, $\alpha_2\delta$-$1^{+/+}$ (left panel, repeated from Fig. 2 for comparison) and $Ca_V2.2\_HA^{KI/KI}$, $\alpha_2\delta$-$1^{-/-}$ (right panel) mice. Sections are always oriented as contra (left) and ipsi (right) to PSNL. Sections were stained for $Ca_V2.2$_HA (i, v; green), CGRP (ii, vi; magenta) and IB4 (iii, vii; red). Bottom panel (iv, viii) shows merged images. Open arrows, areas of decreased staining in superficial dorsal horn. closed arrows, area of increased $Ca_V2.2$_HA staining in medial deep dorsal horn. Scale bar: 200 µm

B, C: Quantification of immunofluorescence for IB4 (B) and CGRP (C) in superficial dorsal horn (Laminae I and II, from pial surface to 80 µm) from the medial, central and lateral ROIs in $Ca_V2.2\_HA^{KI/KI}$, $\alpha_2\delta$-$1^{+/+}$ (WT, left column) and $Ca_V2.2\_HA^{KI/KI}$, $\alpha_2\delta$-$1^{-/-}$ (KO, right column) mice. Data are represented as ratio of immunofluorescence (ipsi/contra to PSNL) and individual symbols represent the data for each animal; black bars show mean ± s.e.m (for WT, N = 5 mice, 35 sections (WT); for KO, N = 7 mice, 49 sections). The individual *P* values for Medial, Central and Lateral regions are: 0.1315, 0.1500 and 0.1739 for WT and 0.0246, 0.0001 and 0.0107 for KO, respectively in B: and 0.2176, 0.5092 and 0.7585 for WT and 0.8882, 0.2893 and 0.8758 for KO, respectively in C. (* $P < 0.05$, ** $P < 0.01$, *** $P < 0.001$)

**Supplementary Figure 3: High resolution analysis of $Ca_V$2.2_HA, IB4 and CGRP clusters in dorsal horn of $Ca_V$2.2_HA$^{KI/KI}$, $\alpha_2\delta$-1$^{-/-}$ PSNL mice.**

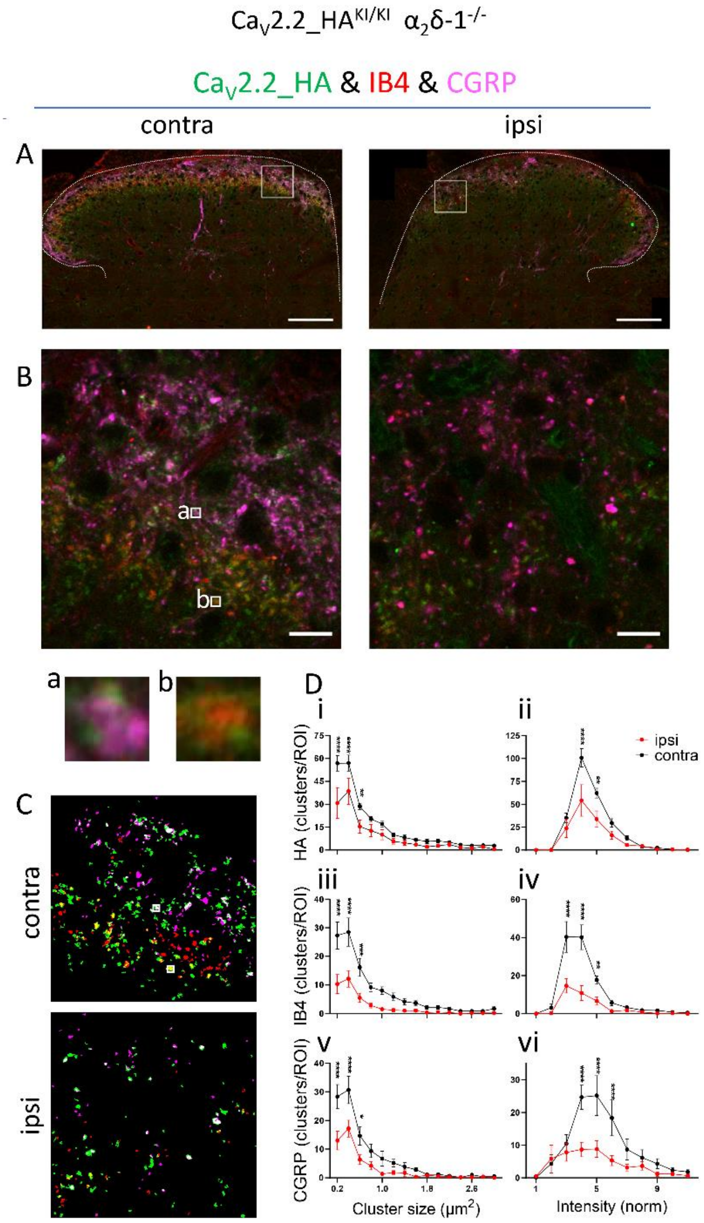


A: Representative Airyscan tiled images of contra (left) and ipsi (right) dorsal horn from the same sections following PSNL stained for $Ca_V$2.2_HA (green), IB4 (red) and CGRP (magenta). An ROI of 70 x 70 µm placed in the medial region of the superficial layer for quantification of clusters is indicated in A, and shown enlarged in B. Representative examples of CGRP (a) and IB4 (b) positive glomeruli are indicated by small ROIs of 2 x 2 µm on the contra side of B and enlarged underneath. Scale bars in A and B; 100 µm and 10 µm respectively.

C: Composite mask from the 3 channels ($Ca_V$2.2_HA (green), IB4 (red) and CGRP (magenta)) from ROIs shown in B, for particles between 0.2-5 µm$^2$ with signal above threshold (see Methods).

D: Size (i, iii, v) and intensity (ii, iv, vi) distribution of clusters positive for $Ca_V$2.2_HA, IB4 and CGRP for the ipsi (red) and contra (black) side in the medial superficial ROI. N: 840, 228 and 292 (ipsi) and 1491,706 and 656 (contra) for $Ca_V$2.2_HA, IB4 and CGRP, respectively. Data from 12 medial superficial ROIs (6 contra & 6 ipsi), from 3 sections from each of 2 mice. Statistical significance of differences denoted by * $P < 0.05$, ** $P < 0.01$, *** $P < 0.001$, **** $P < 0.0001$. Details of statistical test results (Repeated-measures 2-way ANOVA followed by Šídák's multiple comparisons test) are in Supplementary Information.

**Supplementary Figure 4: Comparison of DRG neurons immunoreactive for GFRα1 ipsilateral and contralateral to PSNL in $\alpha_2\delta$-1$^{+/+}$ and $\alpha_2\delta$-1$^{-/-}$DRGs**

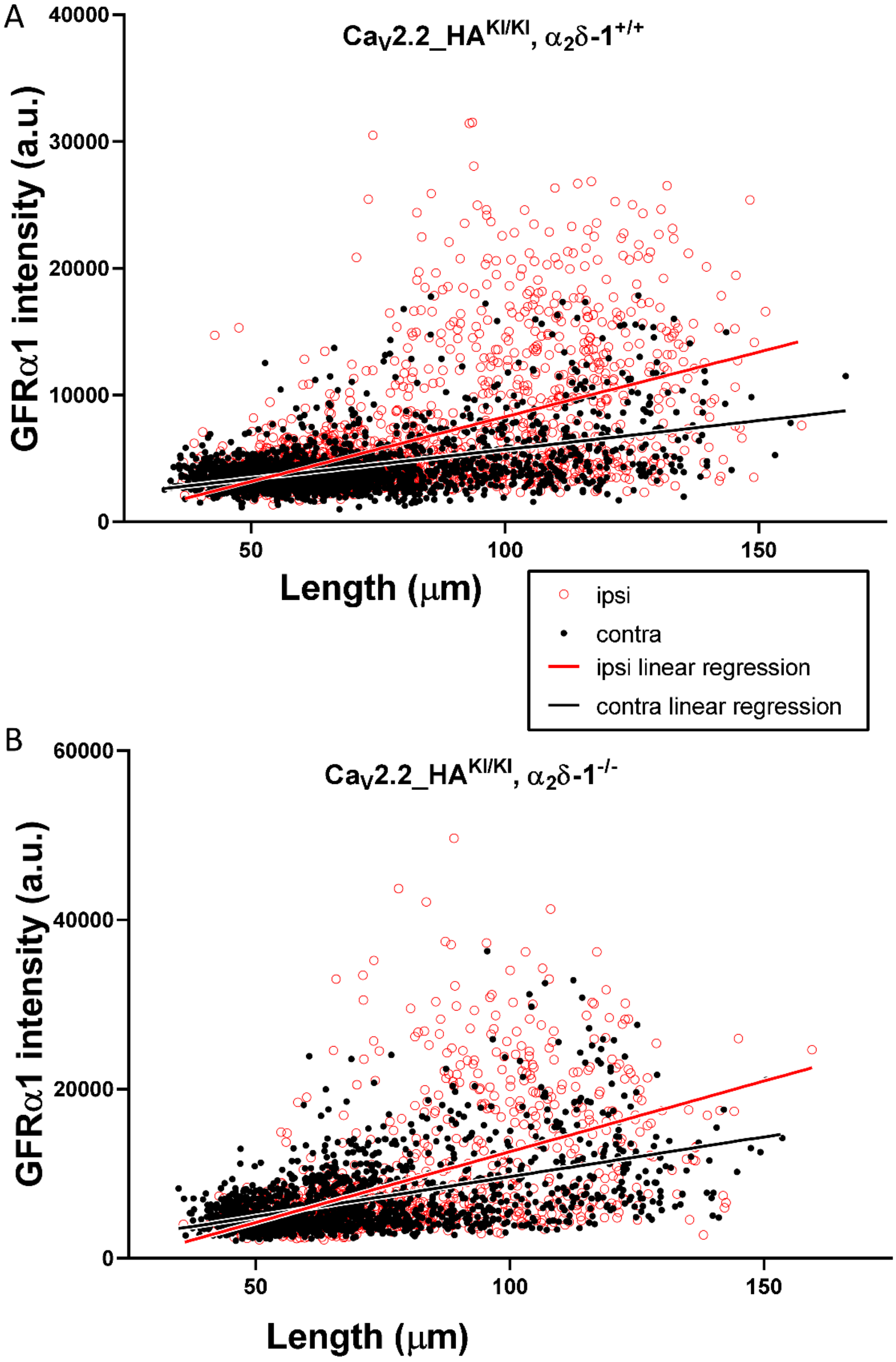


GFRα1 immunofluorescence (a.u) of all DRG neurons plotted with respect to their perimeter (μm), from (A) $Ca_V$2.2_HA$^{KI/KI}$, $\alpha_2\delta$-1$^{+/+}$ (n=2108 ipsi to PSNL (open red symbols), and 2149 contra cells (solid black symbols) from 4 mice) and (B) $Ca_V$2.2_HA$^{KI/KI}$, $\alpha_2\delta$-1$^{-/-}$ (n=1091 ipsi to PSNL (open red circles) and 1481 contra (solid black circles) cells from 3 mice). The fitted linear regression lines are in red (ipsi) and black (contra). For (A), the slopes 102.2 ± 3.5 (ipsi) and 45.8 ± 1.8 (contra) are significantly different, $F_{(1,4256)} = 207.1$, $P<0.0001$. For (B), slopes are 183.6 ± 9.5 (ipsi), 89.3 ± 4.1 (contra) are significantly different, $F_{(1,2571)} = 101.1$, $P<0.0001$.

**Supplementary Figure 5: Analysis of combined $Ca_V$2.2_HA cluster data for deep dorsal horn ROIs**

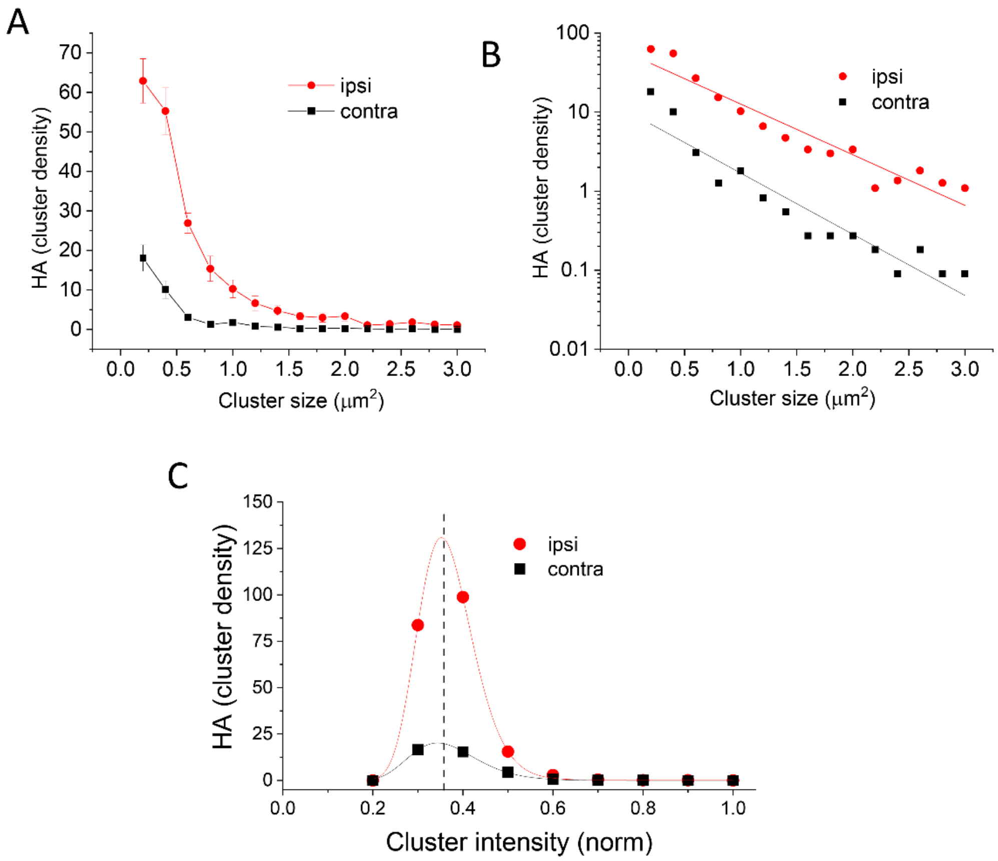


A-C: Combined cluster analysis for all $Ca_V$2.2_HA data from medial deep dorsal horn, for cluster size (A, B) and cluster intensity (C), ipsi (red) and contra (black) to PSNL. The data are combined from experiments from Fig. 3 (deep dorsal horn ROIs not shown) and Fig. 8. Data for cluster size (A) are re-plotted on a log scale in (B) and fit by linear regression with slopes of 0.64 ± 0.05 (ipsi) and 0.77 ± 0.07 (contra), which are not statistically different. Data for cluster intensity (C) are fit with a log normal distribution with midpoint 0.360 ± 0.002 (ipsi) and 0.362 ± 0.001 (contra).

**Supplementary Figure 6 High resolution analysis of $Ca_V$2.2_HA, and GFRα1 clusters in superficial dorsal horn following PSNL.**

**Supplementary text** Contralateral to PSNL, analysis showed 7.0 % of $Ca_V$2.2_HA glomerular clusters were associated with GFRα1, and 8.9 % of GFRα1 clusters were associated with $Ca_V$2.2_HA (Supplementary Fig. 6A i and enlarged images in B; association determined from 2878 $Ca_V$2.2_HA clusters and 2388 GFRα1 clusters from the combined 3 ROIs from each side of each section, for 6 sections from 2 mice). Ipsilateral to PSNL, there was a marked reduction of $Ca_V$2.2_HA clusters (1477) in agreement with our previous results, but a very similar number of GFRα1 clusters (2338). Of these, 5.8 % of $Ca_V$2.2_HA clusters were associated with GFRα1 and only 3.9 % of GFRα1 clusters showed association with $Ca_V$2.2_HA. We also compared the density in the superficial medial ROIs (number of clusters / total ROI area) and intensity of $Ca_V$2.2_HA and GFRα1 clusters, from the masks in Supplementary Fig. 6A. As we found for the analysis in Fig. 3, only the density of $Ca_V$2.2_HA clusters decreased in the superficial dorsal horn ipsilateral to PSNL (Supplementary Fig. 6C, i and ii), with no change in size profile (Supplementary Fig. 6C, i) or intensity distribution (Supplementary Fig. 6C, ii). In contrast, the density of GFRα1 clusters was increased in the superficial dorsal horn, ipsilateral to PSNL (Supplementary Fig. 6C, iii and iv). However, there was no change in size profile (Supplementary Fig. 6C, iii) or intensity distribution (Supplementary Fig. 6C, iv) of the GFRα1 clusters.

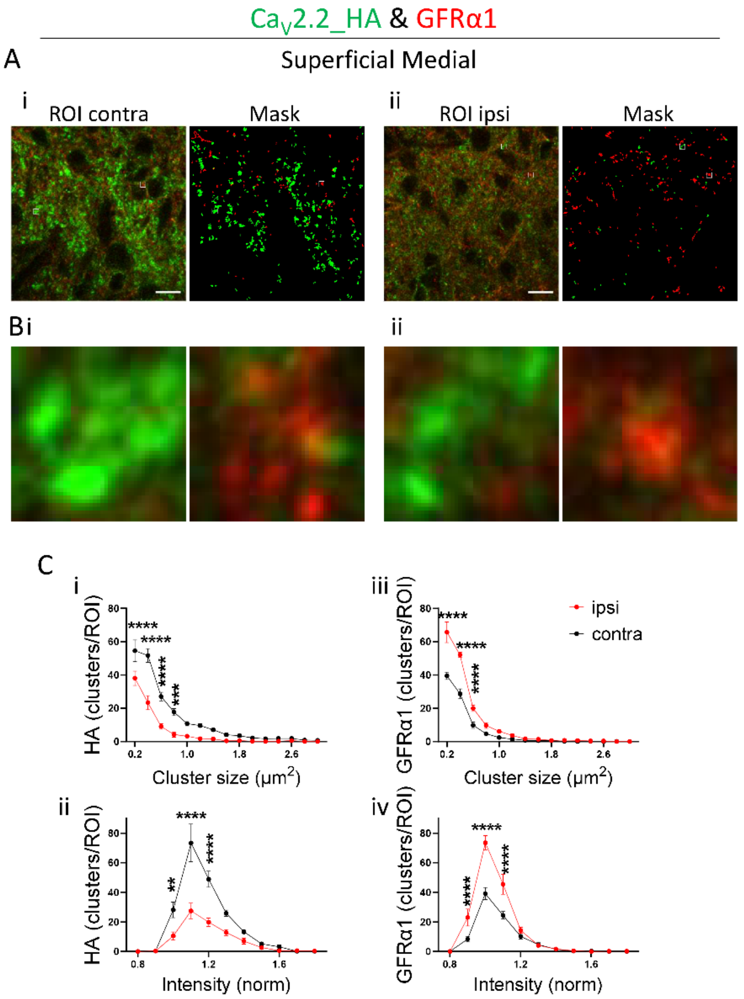


**Figure legend**

A: Airyscan images from ROIs (70 x 70 μm, with dotted lines in Fig. 7A) in the medial region of the superficial contra (i) and ipsi (ii) dorsal horn from the same section following PSNL stained for $Ca_V$2.2_HA (green) and GFRα1 (red). Corresponding composite mask from the 2 channels, used for cluster analysis with $Ca_V$2.2_HA

(green) and GFRα1 (red) is shown on the right of each ROI, with signal above threshold and particles between 0.2 - 5 $\mu m^2$ (see Methods). Scale bars 10 µm.

B: Enlarged 2 x2 µm ROIs containing $Ca_V2.2$_HA or GFRα1 positive clusters contra (i) and ipsi (ii) to PSNL.

C: Size (i, iii) and intensity (ii, iv) distribution of clusters positive for $Ca_V2.2$_HA (i, ii) and GFRα1 (iii, iv) ipsi (red) and contra (black) to PSNL in medial superficial ROIs. N = 497 $Ca_V2.2$_HA and 980 GFRα1 clusters ipsi, and 1194 $Ca_V2.2$_HA and 536 GFRα1 clusters contra to PSNL. Data from 24 ROIs (6 superficial medial ROIs from each side), from 3 section per each of 2 mice. Statistical significances are denoted by * $P < 0.05$, ** $P < 0.01$, *** $P < 0.001$, **** $P < 0.0001$. Details of statistical test results (Repeated-measures 2-way ANOVA followed by Šídák's multiple comparisons test) are in Supplementary Information.

**Supplementary Figure 7: High resolution analysis of $Ca_V2.2$_HA, and GFRα1 clusters in dorsal horn of $Ca_V2.2$_HA$^{KI/KI}$, $\alpha_2\delta$-1$^{-/-}$ PSNL mice.**

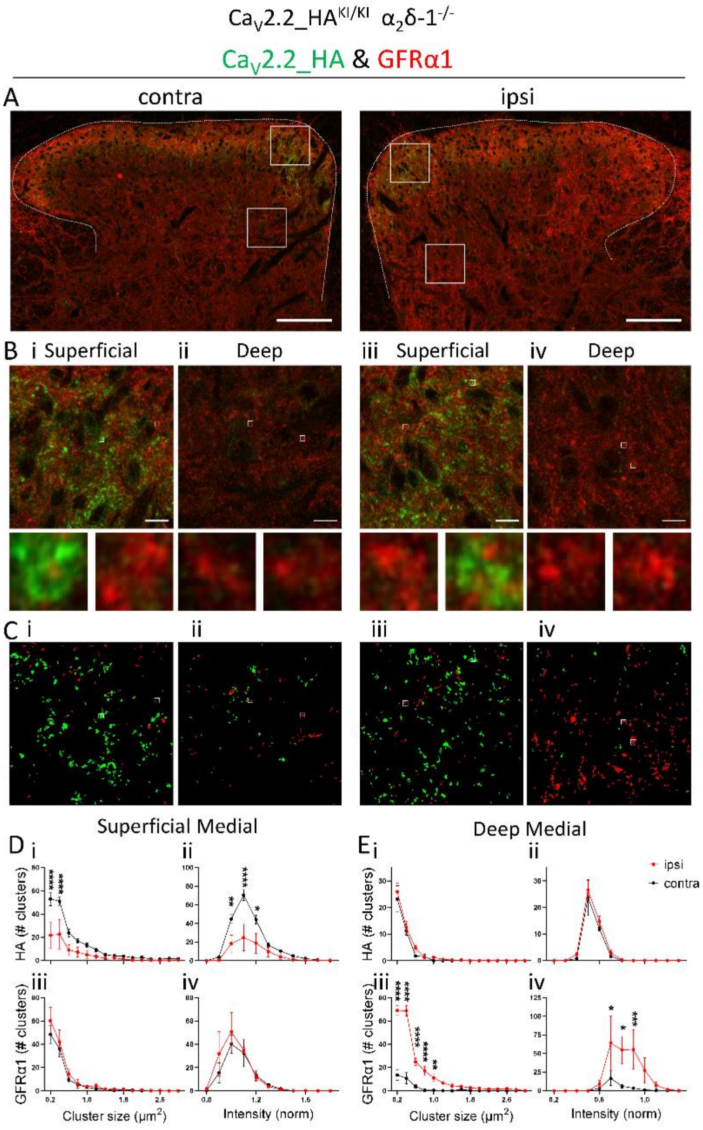


A, B: Representative Airyscan tiled images (A) of contra (left) and ipsi (right) dorsal horn from the same sections following PSNL stained for $Ca_V2.2$_HA (green), and GFRα1 (red). Medial ROI (70 x 70 µm) in the medial region of the superficial (i and iii) and deep (ii and iv) layers of each side, used for cluster analysis are indicated with squares in (A), and shown enlarged in (B). Two representative example ROIs (2 x2 µm) containing $Ca_V2.2$_HA or GFRα1 positive clusters are enlarged underneath each panel. Scale bars in A and B; 100 µm and 10 µm respectively.

C: Corresponding composite masks from the 2 channels, $Ca_V2.2$_HA (green) and GFRα1 (red) from ROIs shown in B, with particles between 0.2 - 5 µm$^2$ with signal above threshold (see Methods).

D, E: Size (i, iii) and intensity (ii, iv) distribution of clusters positive for $Ca_V2.2$_HA and GFRα1 in the ipsi (red) and contra (black) side of the medial superficial (D) and deep (E) ROIs. (D) In the superficial layers N = 313 and 543 (ipsi) and 793 and 433 (contra) $Ca_V2.2$_HA and GFRα1 positive clusters, respectively. (E) In the deep layer N = 187 and 878 (ipsi) or 153 and 125 (contra) $Ca_V2.2$_HA and GFRα1 positive clusters respectively. 16 ROIs were analysed (4 medial superficial ROIs and 4 deep ROIs from each side, from 2 section per each of 2 mice). Statistical significance of differences are denoted by * $P < 0.05$, ** $P < 0.01$, *** $P < 0.001$, **** $P <$ 0.0001. Details of statistical test results (Repeated-measures 2-way ANOVA followed by Šídák's multiple comparisons test) are in Supplementary Information.
